# Supplementary material for: Wakefulness Is Governed by GABA and Histamine Cotransmission
Source: Neuron. 2015 Jul 1;87(1):164–78. doi: 10.1016/j.neuron.2015.06.003 (PMC4509551; doi:10.1016/j.neuron.2015.06.003)
Supplement: Document S1. Figures S1–S6 and Supplemental Experimental Procedures [file mmc1.pdf]

Neuron, Volume *87*

Supplemental Information

Wakefulness Is Governed by GABA and Histamine Cotransmission

Xiao Yu, Zhiwen Ye, Catriona M. Houston, Anna Y. Zecharia, Ying Ma, Zhe Zhang, David S. Uygun, Susan Parker, Alexei L. Vyssotski, Raquel Yustos, Nicholas P. Franks, Stephen G. Brickley, and William Wisden

Supplementary Items

Figure S1 (related to Figure 3) shows the striatum-based turning assay used to test the efficacy of AAV constructs that knockdown or delete vGAT in striatum.

Figure S2 (related to Figures 3 and 4) shows the extent of *AAV-Cre-2A-Venus* expression in the TMN area resulting from bilateral injections.

Figure S3 (related to Figure 3F) shows qPCR on mRNA isolated from TMN tissue punches or single cells of *HDC-vgat KD* and *TMN-Δvgat* mice to assay the knockdown or deletion efficiency of *vgat* expression.

Figure S4 (related to Figures 3 and Figure 4) shows histamine levels in the neocortex and striatum of *HDC-vgat KD* and *TMN-Δvgat* mice.

Figure S5 (related to Figure 4) shows examples of EEG and EMG data used for scoring vigilance states and the time mice spent in each state.

Figure S6 (related to Figure 4) shows how the *HDC-vgat KD* and *TMN-Δvgat* mice responded to sleep deprivation.

Experimental Procedures

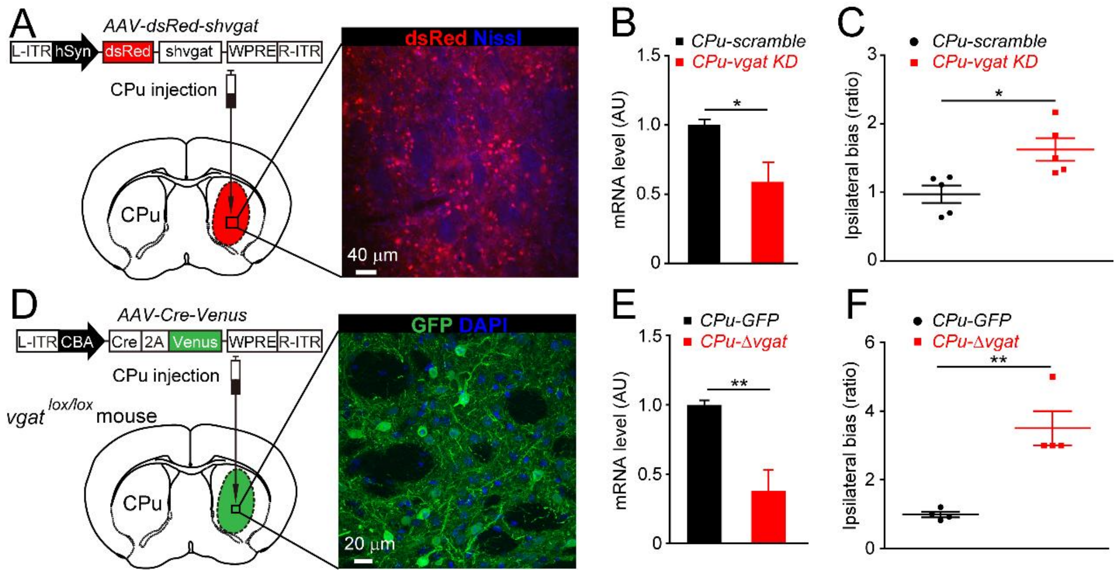

Figure S1 Striatal-based motor turning to check efficacy of the AAV constructs that knockdown or delete *vgat* expression in striatum. *Vgat* shRNA knockdown in the CPu was as effective as unilateral ablation of the *vgat* gene. Both the *CPu-vgat KD* and *CPu-Δvgat* mice had increased ipsilateral turning compared with controls.

(A) *AAV-dsRed-shvgat* and *AAV-dsRed-scramble* were unilaterally injected into the caudate putamen (CPu) of adult C57Bl6 mice to make *CPu-vgat KD* and *CPu-scramble* mice, respectively. Four weeks after injection, dsRed expression was visible in the CPu. Background: the CPu contains mainly GABAergic (medium spiny) projection neurons. When striatal function is unilaterally perturbed, animals tend to turn predominantly in one direction. (B) Q-PCR of *vgat* gene expression from CPu tissue punches shows *AAV-dsRed-shvgat* injection decreased *vgat* transcript levels compared with *AAV-dsRed-scramble*-injected control mice (*CPu-scramble*: 1 ± 0.04 *vs. CPu-vgat KD*: 0.58 ± 0.14, t-test, *p < 0.05). (C) Turning behavior experiments were performed with a separate cohort of injected mice. Knocking down *vgat* expression by injecting ipsilaterally *AAV-dsRed-shvgat* into the CPu caused

ipsilateral turning bias in those animals. Error bars represent sem. (*CPu-scramble*: 0.97 ± 0.12*, vs. CPu-vgat KD*: 1.62 ± 0.16*,* t-test, *p < 0.05). Method: a camera was attached above an open box, and mice were put into the box for 30 min. Data were collected by counting the video for the first 1 min or 5 min. (D) *AAV-Cre-2A-Venus* or *AAV-GFP* was injected into the CPu of $vgat^{lox/lox}$ mice to make *CPu-Δvgat* and *CPu-GFP* mice, respectively. Four weeks after injection, Venus was expressed in the CPu as seen by staining with GFP antisera. (E) qPCR of *vgat* gene expression from a virus injected CPu punch showed *AAV-Cre-Venus* decreased *vgat* gene expression compared with *AAV-GFP* injected control mice (*CPu-GFP*: 1 ± 0.03 *vs. CPu-Δvgat*: 0.37 ± 0.15, t-test, **p < 0.01, Error bars, sem). (F) Locally knocking out the *vgat* gene by injecting *AAV-Cre-2A-Venus* ipsilaterally into a portion of the CPu of $vgat^{lox/lox}$ mice caused an ipsilateral turning bias in those animals (*CPu-GFP*: 0.99 ± 0.08 *vs. CPu-Δvgat*: 3.5 ± 0.5, t-test, **p < 0.01).

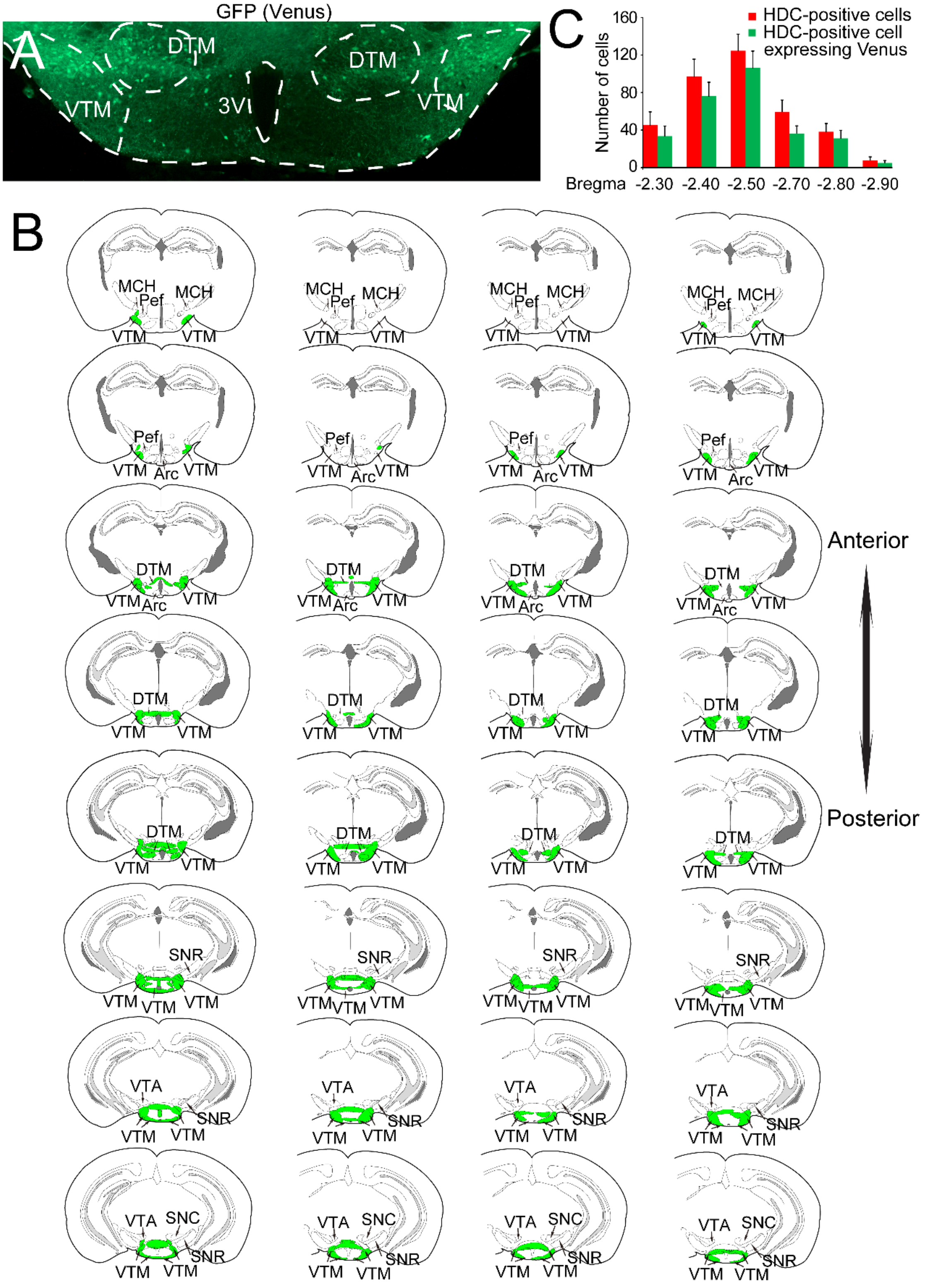

Figure S2 Mapping *AAV-Cre-2A-Venus* expression in the TMN area of *vgat*$^{lox/lox}$ mice (*TMN-Δvgat* mice). (A) Coronal section with bilateral *AAV-Cre-2A-Venus* injection shows Venus (GFP) expression in the VTM and DTM

area of the TMN. (B) Illustration of AAV transgene expression from 4 mice. Whole-brain sections were incubated with GFP antisera. GFP expression in soma was observed (as shown in A) and illustrated with green shading. Most of the GFP positive cells were restricted to the TMN area. A few GFP-cells were observed in the Pef area (n = 5 ± 2 cells; n = 4 mice) and in the substantia nigra reticulata (SNR) area (n = 4 ± 2 cells; n = 4 mice). Error bars, sem. (C) All brain sections were stained with HDC and GFP antisera and the HDC-positive and GFP-positive cells were counted. Most HDC-positive neurons were transduced by *AAV-Cre-2A-Venus* as determined by GFP expression (77 ± 2 %, n = 4 mice). Error bars, sem.

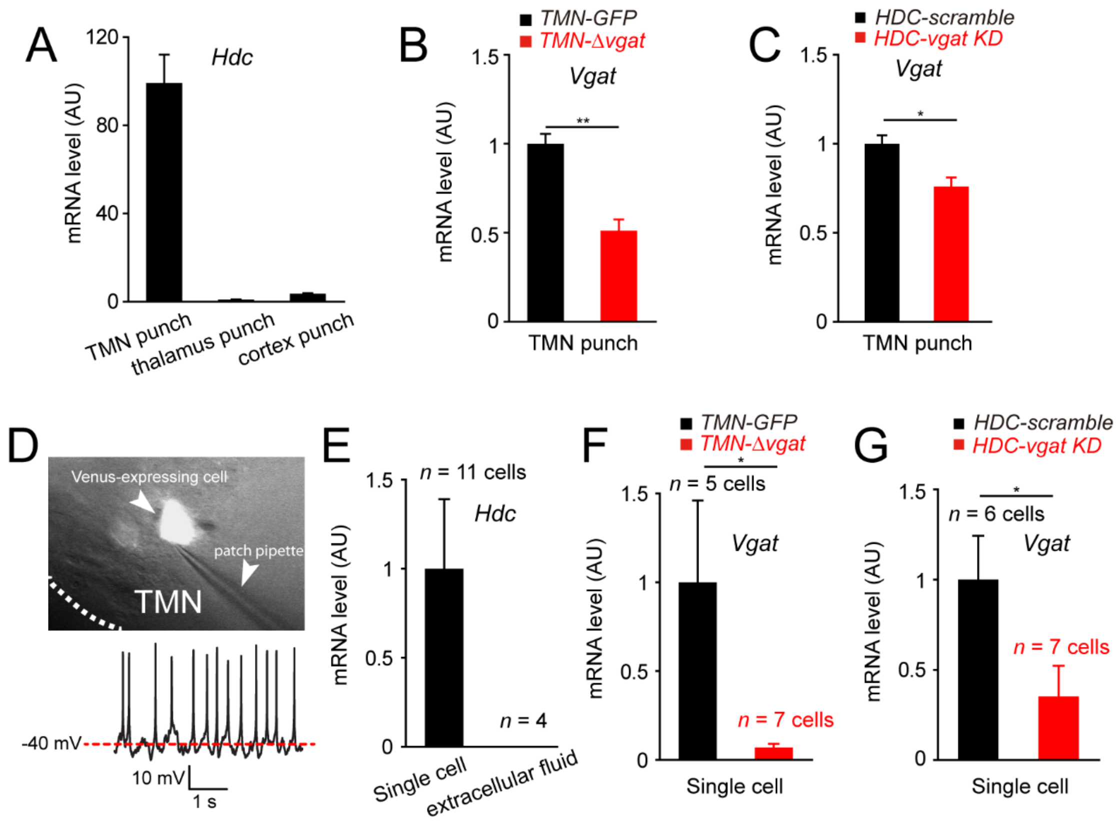

Figure S3 Testing efficiencies of deleting or knocking-down *vgat* expression in the TMN area of *TMN-Δvgat* and *HDC-vgat KD* mice. (A) positive control: qPCR on mRNA isolated from tissue punches from the TMN area showed that *hdc* was highly expressed in TMN punches, but not in thalamus and neocortex punches (*n* = 4 mice). (B, C) *vgat* mRNA levels were significantly decreased in the TMN punches of *TMN-Δvgat* (*n* = 4) and *HDC-vgat KD* (*n* = 4) mice compared with those from *TMN-GFP* (*n* = 4) and *HDC-scramble* (*n* = 4) mice (t-test, *p < 0.05, **p < 0.01). (D) An example of a Venus-expressing TMN cell from an acute brain slice of TMN from a *HDC-Δvgat* mouse. The neuron was patched and its firing (4 Hz) recorded. The patch pipette is visible. (E) Single-cell qPCR of *hdc* mRNA in some selected patched cells. *Hdc* mRNA was detected in patched TMN cells but not in extracellular fluid. In *HDC-scramble* and *HDC-vgat KD* mice, all the patched

cells were *hdc*-positive (13 out of 13 cells), because the fluorescence from AAV transduction is *HDC-Cre* dependent; in *TMN-Δvgat* and *TMN-GFP* mice, only 12 out of 16 cells patched were *hdc*-positive due to non-cell-type selective expression of the fluorescent AAV transduction (Cre-2A-Venus). (F, G) *vgat* mRNA levels were significantly decreased in the single patched cells from the TMN area of *TMN-Δvgat* (*n* = 7 cells) and *HDC-vgat KD* (*n* = 7 cells) mice compared with cells from *TMN-GFP* (*n* = 5 cells) and *HDC-scramble* (*n* = 6 cells) mice (t-test, *p < 0.05). Error bars, sem.

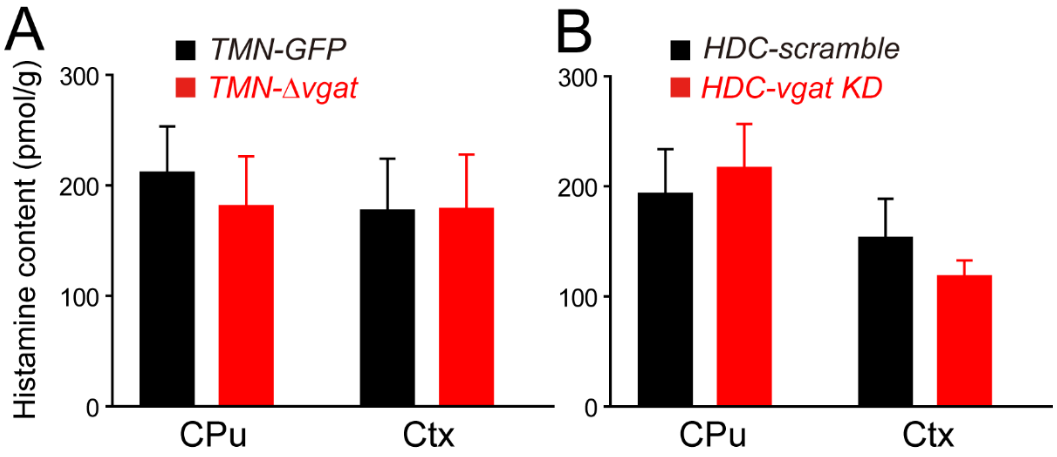

Figure S4 Histamine level in the neocortex or striatum of *HDC-vgat KD* and *TMN-Δvgat* mice. (A) Histamine levels in the striatum or neocortex of *TMN-GFP* (*n* = 6) and *TMN Δvgat* (*n* = 5) mice (t-test, $p > 0.05$, bars, sem). (B) Histamine levels in the striatum or neocortex of *HDC-scramble* (*n* = 5) and *HDC-vgat KD* (*n* = 4) mice (t-test, $p > 0.05$, bars, sem).

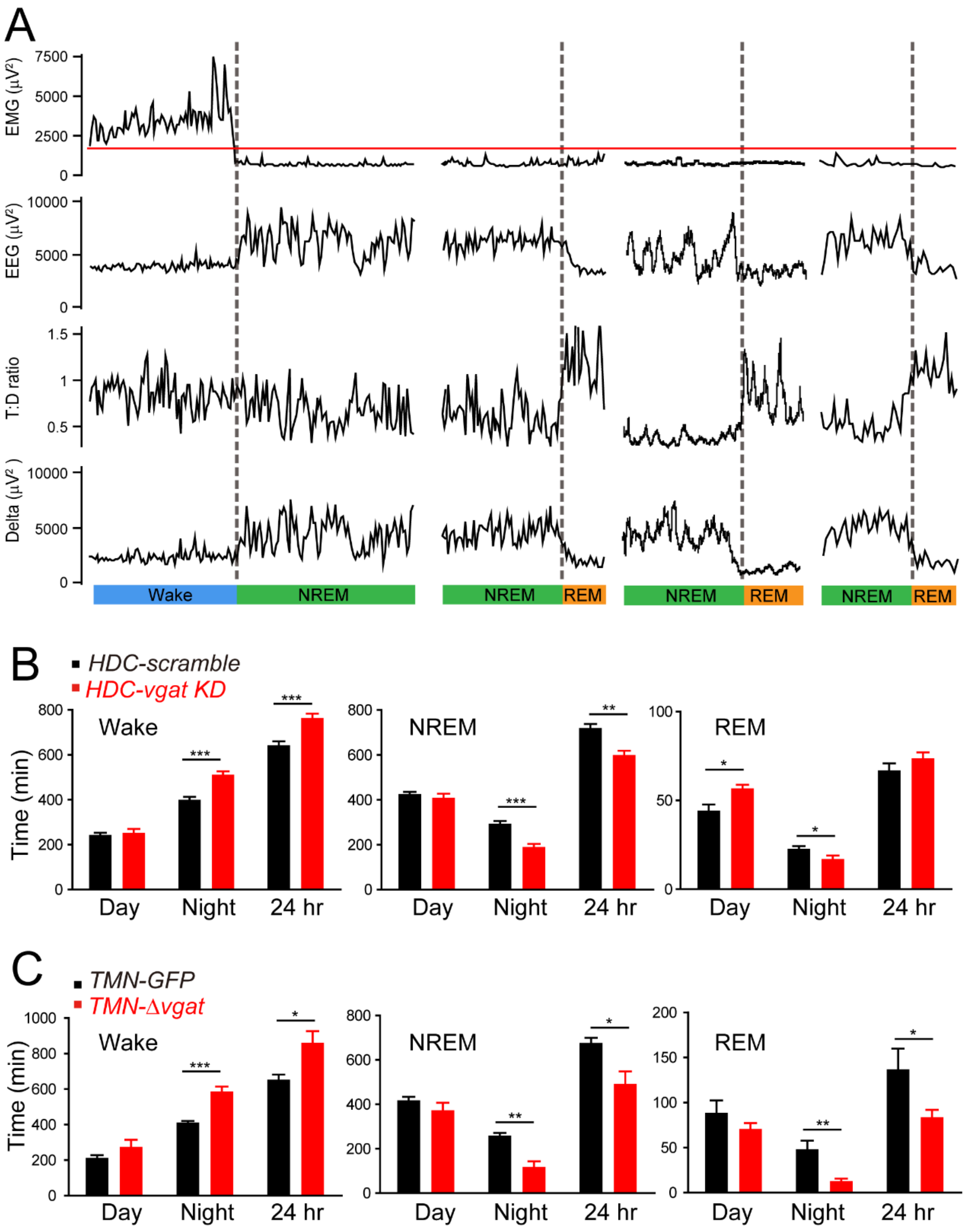

Figure S5 EEG scoring and additional sleep-wake parameters from *HDC-vgat KD* and *TMN-Δvgat* mice. (A) Example traces of EMG and EEG power during wake, NREM, and REM from the mice. T:D ratio, theta/delta ratio. (B) Time of wake, NREM and REM sleep during the day (0-12 h), night (12-24 h) and entire 24 hr of *HDC-scramble* (*n* = 6) and *HDC-vgat KD* (*n* = 6) mice (t-test, *p < 0.05, **p < 0.01, ***p < 0.001, bars, sem). (C) Time of wake, NREM

and REM sleep during the day (0-12 h), night (12-24 h) and entire 24 hr of *TMN-GFP* (n = 5) and *TMN-Δvgat* (n = 7) mice (t-test, *$p < 0.05$, **$p < 0.01$, ***$p < 0.001$, bars, sem).

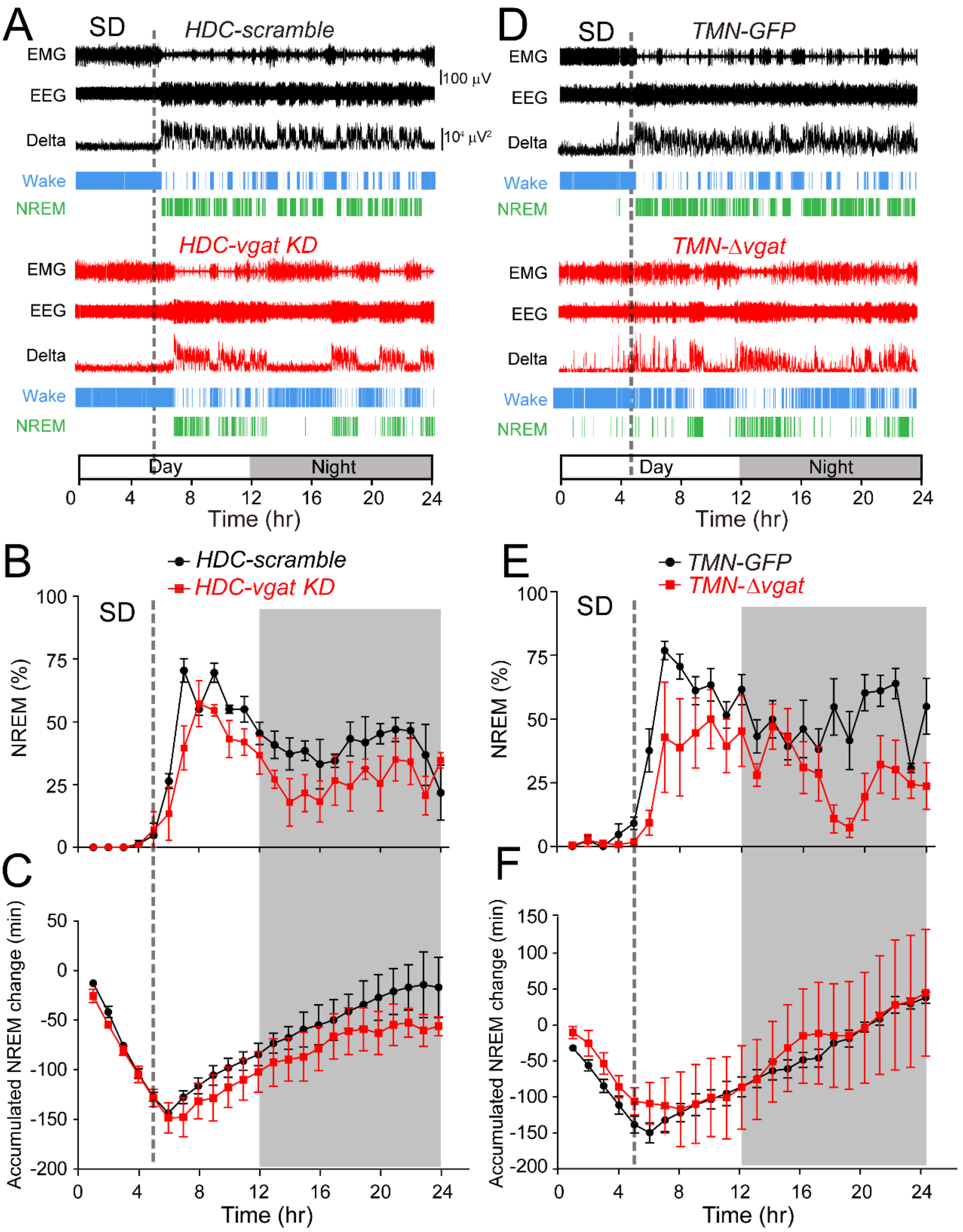

Figure S6. Responses to sleep deprivation of *HDC-vgat KD* and *TMN-Δvgat* mice. (A) Continuous EMG, EEG, delta power, wake, NREM sleep and REM sleep scoring data recorded for an *HDC-scramble* and an *HDC-vgat KD* mouse during and after sleep deprivation (SD). The period where SD ends is marked by the perpendicular dotted gray line. (B) Percentage of NREM sleep

during 5-hr sleep deprivation and following 19-hr recovery sleep of *HDC-scramble* (*n* = 4) and *HDC-vgat KD* (*n* = 4) mice. Bars, sem. (C) The accumulated NREM change compared with the normal 24-hr sleep-wake cycle. The proportional loss and recovery of NREM sleep in minutes during and after sleep deprivation of *HDC-scramble* (*n* = 4) and *HDC-vgat KD* (*n* = 4) mice. Bars, sem. (D) Continuous EMG, EEG, delta power, wake, NREM sleep and REM sleep scoring data recorded for a *TMN-GFP* and *TMN-Δvgat* mouse during and after sleep deprivation (SD). The period where SD ends is marked by the perpendicular dotted gray line. (E) Percentage of NREM sleep during 5-hr sleep deprivation and following 19-hr recovery sleep of *TMN-GFP* (*n* = 5) and *TMN-Δvgat* (*n* = 4) mice. Bars, sem. (F) The accumulated NREM change compared with the normal 24-hr sleep-wake cycle. The proportional loss and recovery of NREM sleep in minutes during and after sleep deprivation of *TMN-GFP* (*n* = 5) and *TMN-Δvgat* (*n* = 4) mice. Bars, sem.

Experimental Procedures

Mouse genetics

To generate the *HDC-YFP* mice, *HDC-Cre* mice were crossed with *Rosa26-loxP-Stop-loxP-YFP* mice. To generate the *vGAT-tdTomato* mice, *vGAT-Cre* mice were crossed with *Rosa26-loxP-Stop-loxP-tdTomato* mice. Genotyping primers were:

***iCre* (*HDC-Cre*)**:

forward: 5'GTGTGGCTGCCCCTTCTGCC3',

reverse: 5'AGCCTCACCATGGCCCCAGT3' (250 bp product);

vgat$^{lox/lox}$:

common: 5'TCCTTTGTGGCTTCCTTCCG3',

WT reverse: 5'GGATAGAAGAAGTGTGGACC3',

Mut reverse: 5'GCAGTGGACCTTGGATGTCTATC3' (WT: 185 bp, Mut: 250 bp);

Rosa-YFP:

forward:5'GGCGCTACCGGTCGCCACCATGGTGAGCAAGGGCGAGGAGC 3',

reverse: 5'GCGCGCGTTAACTTACTTGTACAGCTCGTCCATGCC3' (980 bp);

vGAT-Cre:

forward: 5'AGCGATGGATTTCCGTCTCT3',

reverse: 5'CACCAGCTTGCATGATCTCC3' (200 bp)

Rosa-tdTomato:

WT forward: 5'AGGGGAGCTGCAGTGGAGTA3',

WT reverse: 5'CCGAAAATCTGTGGGAAGTC3';

Mut forward: 5'GGCATTAAAGCAGCGTATCC3',

Mut reverse: 5'CTGTTCCTGTACGGCATGG3' (WT: 297 bp, Mut: 196 bp).

Design and testing of *shvgat* expression vectors

Hairpin (*sh*) oligonucleotides were designed online (http://katahdin.mssm.edu/siRNA/RNAi.cgi?type=shRNA). The *sh* oligonucleotide sequences tested were:

scramble: 5'-TGCTGTTGACAGTGAGCGGCCGCGATTAGGCTGTTATAATAGTGAAGCCACAGATGTATTATAACAGCCTAATCGCGGCTGCCTACTGCCTCGGA-3'

shvgat1: 5'-TGCTGTTGACAGTGAGCGCGGTGTGCTCGTGGTGAATAAGTAGTGAAGCCACAGATGTACTTATTCACCACGAGCACACCATGCCTACTGCCTCGGA-3'

shvgat2: 5'-TGCTGTTGACAGTGAGCGAAGCCATTCAGTGCTTGGAATCTAGTGACCACAGATGTAGATTCCAAGCACTGAATGGCTGTGCCTACTGCCTCGGA-3'

shvagt3: 5'-TGCTGTTGACAGTGAGCGCACTCATCTTGTGCAATGTATCTAGTGAAGCCACAGATGTAGATACATTGCACAAGATGAGTTTGCCTACTGCCTCGGA-3'

The *sh*-oligonucleotides were amplified by VENT polymerase (NEB, UK) using primers with added XhoI and EcoRI sites underlined (forward: 5'-GATGGCTG-CTCGAG-AAGGTATAT-TGCTGTTGACAGTGAGCG-3'; reverse: 5'-GTCTAGAG-GAATTC-CGAGGCAGTAGGCA-3'). Products were cloned into *the EcoRI/XhoI* site of the pPRIME-CMV-dsRed-FF3 vector (Stegmeier et al., 2005) to generate *pPRIME-dsRed-scramble*, *pPRIME-dsRed-shvgat1* (oligo 1), *pPRIME-dsRed-shvgat2* (oligo 2) and *pPRIME-dsRed-shvgat3* (oligo 3) (Figure 3A). The *pPRIME-CMV-dsRed-FF3* vector was a gift from Stephen Elledge (Addgene plasmid 11664).

To construct the *vgat* expression vector, the mouse *vgat* coding sequence and 3' UTR was PCR amplified with primers (forward: 5'-ATATAT-GCTAGC-CCCAGACCCTTCTGTCCTTTTCTC-3'; reverse: 5'-ATATAT-AAGCTT-CGCCTTTGTTTCTTCTTTATTTGCGG-3') with *HindIII* and *NheI* sites from a mouse *vgat* cDNA clone (Clontech, CA). The digested PCR insert was purified and cloned into the *pcDNA3.1* expression vector. The *vgat* expression plasmid was co-transfected into HEK293 cells with either *pPRIME-dsRed-shvgat1, pPRIME-dsRed-shvgat2, pPRIME-dsRed-shvgat3 or pPRIME-dsRed-scramble*. 24-36 hours after transfection, cells were fixed with paraformaldehyde, and stained with vGAT antisera (see section "Immunocytochemistry" below); dsRed was visualized by primary fluorescence. Using this method, we established that *shvgat* oligo3 was

effective. The *shvgat* and *scramble* segments were then placed into AAV genomes (see next section).

To generate the *AAV-dsRed-scramble* and *AAV-dsRed-shvgat* transgenes, the *pPRIME-dsRed-scramble* and *pPRIME-dsRed-shvgat* vectors were PCR amplified using the primers (forward: 5'-AGTGAACCGTCGGCGCGCCTAGCGCTACC-3'; reverse: 5'-CGTGTTTAAACGCGCTAGCCTTCCAATTG-3') with an *AscI* site in the forward primer and a *NheI* site in the reverse primer. PCR products, and were digested with *AscI* and *NheI*, and cloned into an AAV vector with a human synapsin promoter. To construct *AAV-flex-dsRed-scramble* and *AAV-flex-dsRed-shvgat* plasmids, *pPRIME-dsRed-scramble* and *pPRIME-dsRed-shvgat* were PCR amplified using primers (forward: 5'-GAACCGTCAGATCCGCTAGCGCTACCG-3'; reverse: 5'-GAACGCGTCGGCGCGCCTTTAAACGCAT-3') with a *NheI* site in the forward primer and an *AscI* site in the reverse primer. PCR products and AAV vector were digested with *AscI* and *NheI*, and cloned into the AAV vector. The AAV transgenes were then packaged into capsids (see Experimental procedures in the main manuscript).

Histology

For immunocytochemistry, mice were transcardially perfused with 4% paraformaldehyde in phosphate buffered saline (PBS). Brains were removed and 35-μm-thick coronal sections were cut using a Leica VT1000S vibratome. Free-floating sections were washed in PBS three times for 5 min, permeabilized in PBS plus 0.4% Triton X-100 for 30 min, blocked by incubation in PBS plus 5% normal goat serum (NGS) (Vector), 0.2% Triton X-100 for 1 hour (all at room temperature) and subsequently incubated with primary antisera or antibody diluted in PBS plus 2% NGS for 24-48 hours at 4°C on a shaker. Incubated slices were washed three times in PBS for 10 min at room temperature, and then incubated for 2 hours at room temperature with a 1:1000 dilution of a secondary antibody (Molecular Probes) in PBS plus 1% NGS, and subsequently washed there times in PBS for 10 min at room temperature. For cell cultures, medium was removed and cells were washed

in PBS three times for 5 min, and then fixed in PFA for 10 min, after that, cells were permeabilized in PBS plus 0.4% Triton X-100 for 10 min; blocked by incubation in PBS plus 5% normal goat serum (NGS) (Vector), 0.2% Triton X-100 for 10 min (all at room temperature) and subsequently incubated with primary antisera or antibody diluted in PBS plus 2% NGS, shaken overnight at 4°C. Incubated cells were washed three times in PBS for 10 min at room temperature, and then incubated for 2 hours at room temperature with a 1:1000 dilution of a secondary antibody (Molecular Probes) in PBS plus 1% NGS, and subsequently washed there times in PBS for 10 min at room temperature. Primary antisera or antibody used were: rabbit polyclonal EGFP (Invitrogen Molecular Probes, Eugene, OR), 1:1000; guinea pig polyclonal HDC (American Research Products, Belmont, MA), 1:300; mouse monoclonal GAD67 (Millipore, UK), 1:1000; mouse monoclonal vGAT (Synaptic Systems), 1:1000. Secondary antibodies were Alexa Fluor 488 goat anti-rabbit IgG, 1:1000, Alexa Fluor 488 goat anti-mouse IgG, 1:1000; Alexa Fluor 488 goat anti-guinea pig IgG, 1:1000, Alexa Fluor 594 goat anti-rabbit IgG, 1:1000, Alexa Fluor 594 goat anti-mouse IgG, 1:1000; Alexa Fluor 594 goat anti-guinea pig IgG, 1:1000, (Invitrogen Molecular Probes); Slices were mounted on slides, embedded in Mowiol (DAPI or without DAPI), cover-slipped, and analyzed using a Zeiss LSM 510 confocal microscope (Facility for Imaging by Light Microscopy, Imperial College).

Imaging neurons after electrophysiology: Following electrophysiological recording (see section "Electrophysiology"), brain slices were fixed in 4% PFA, permeabilized with 0.2% Triton-X at room temperature for 1-2 hours, and non-specific binding blocked with 5% donkey serum in PBS. A 1:200 dilution of streptavidin-Alexa555 (Invitrogen Molecular Probes) was then conjugated to the biocytin and slices were washed in PBS and mounted in Vectashield® H-1000. Individual neurons were imaged using a Zeiss LSM 510 confocal microscope (Facility for Imaging by Light Microscopy, Imperial College).

EEG analysis and sleep-wake behavior

Surgery was carried out under halothane (1.5–2.5% in oxygen) anesthesia. Three gold-plated EEG electrodes (Decolletage AG) were inserted through

the skull onto the dura mater. For EMG recording, three lengths of Teflon-insulated stainless steel wire were inserted in the neck muscle. Animals were allowed at least 14 days to recover from surgery. Two days before the recording, mice were attached with mock Neurologgers, and then fitted with Neurolgger2A devices. Two EEG and two EMG channels for each mouse were recorded. Spike2 (version 7.10) was used to analysis the sleep (EEG/EMG) data. The sampling rate was set up to 200 Hz. EMG was filtered by band pass between 5 and 45 Hz. EEG frequency was filtered below 0.5 Hz. EEG power was analysed using FFT (Fast Fourier transform of the autocorrelation function) power spectra (FFT size: 1024 or 512).

Sleep deprivation

Mice were attached with mock Neurologgers two days before sleep deprivation. Before sleep deprivation, mice were attached with Neurologger 2A devices. Mice were sleep deprived for 5 hours after ‘‘lights off’’. In the first hour of the sleep deprivation (at the beginning of lights on), mice were put into novel cages with new objects. At each hour, objects were changed with new objects. During the last two hours, mice were gently handled for some seconds when they tended to sleep. Most of the time during the last two hours, mice were active and in a total of two hours, the mice only needed to be handled one or two times. After sleep deprivation, the mice were put back into their home cage to recover.

Electrophysiology

Adult (3-6 months postnatal) mice were handled to reduce their stress, and brain slices were then prepared following cervical dislocation (in accordance with UK Home Office guidelines). The brain was rapidly removed and immersed in ice cold slicing solution. The slicing solution contained (in mM): NaCl 85, KCl 2.5, $CaCl_2$ 0.5, MgCl 3.5, NaH_2PO4 1.25, $NaHCO_3$ 26, D-glucose 11, sucrose 75, 1 kynurenic acid) pH 7.4 when bubbled with 95%O2/5%CO2. Slices were cut using a vibratome tissue slicer (Campden instruments) at a thickness of 250 µm and immediately transferred to a holding chamber containing slicing ACSF continuously bubbled with

95%O2/5%CO2. Once slicing was complete the holding chamber was transferred to a 37°C heat block for 40 minutes, after which the slicing ACSF was gradually exchanged for recording ACSF (in mM: NaCl 125, KCl 2.5, CaCl2 2, MgCl 1, NaH_2PO_4 1.25, $NaHCO_3$ 26, glucose 11, pH 7.4 when bubbled with 95%O2/5%CO2) and allowed to reach room temperature while the solutions were exchanged prior to electrophysiological recording. Slices were visualized using a fixed-stage upright microscope (BX51W1, Olympus and Scientifica Slice scope) fitted with a high numerical aperture water-immersion objective and an infra-red sensitive digital camera. For the optogenetic experiments with *HDC-ChR and HDC-ChR/TMN-Δvgat* mice, we first sliced the TMN area. If there was visible primary fluorescence in this area (e.g. Fig. 6B), we also went on to slice the neocortex and caudate-putamen. Some TMN injections gave no visible primary expression in the TMN and slices from these brains were used as negative controls. A blue (470 nm) collimated LED (M470L3-C1, Thorlabs) was used to illuminate the slice through the objective lens. The LED was driven by an LED-driver (LEDD1B, Thorlabs) which was controlled by a National Instruments digitization board (NI-DAQmx, PCI-6052E; National Instruments, Austin, Texas). The output of the LED was measured with a power meter (PM100D, Thorlabs) positioned below the objective lens and the power output and membrane conductance or voltage changes were aligned for analysis.

Patch pipettes were fabricated from thick-walled borosilicate glass capillaries (1.5 mm outer diameter., 0.86 mm internal diameter, Harvard Apparatus) using a two-step vertical puller (Narishige, PC-10). Pipette resistances were typically 3-4 MΩ when back filled with internal solution. For voltage-clamp experiments, the internal solution contained (in mM): CsCl 140, NaCl 4, $CaCl_2$ 0.5, HEPES 10, EGTA 5, Mg-ATP 2; the pH was adjusted to 7.3 with CsOH. Biocytin (1.5 mg/ml) was included in the pipette solution so that neuronal cell-type could be confirmed. For current-clamp experiments the internal solution contained (in mM): 145 K-gluconate; 4 NaCl; 0.5 $CaCl_2$; 10 HEPES; 5 EGTA; 4 Mg-ATP; 0.3 Na-GTP (adjusted to pH 7.3 with KOH). The amplifier head

stage was connected to an Axopatch 700B amplifier (Molecular Devices; Foster City, CA).

Electrophysiology data analysis

The amplifier current output was filtered at 10 kHz (–3 dB, 8-pole low-pass Bessel) and digitized at 20 kHz using a National Instruments digitization board (NI-DAQmx, PCI-6052E; National Instruments, Austin, Texas). Data acquisition was performed using WINWCP (Version 4.1.2) and WINEDR (Version 3.0.9) kindly provided by John Dempster (University of Strathclyde, UK). The initial rising phase of the spontaneous postsynaptic conductance change (sPSC) was detected on the first derivative of the current record obtained at a command potential of -70 mV. The instantaneous sPSC frequency was calculated from the inter sPSC interval and the running average was calculated every 5 seconds. Cross correlation analysis was undertaken on the timing of PSCs using a 100 ms sliding window before and after each LED pulse. The change in holding current was plotted from the average of each one second current epoch. Any change in the tonic $GABA_A$ receptor meditated conductance (G_{tonic}) was calculated from changes in holding current averaged over at least one minute.

Negative controls for light stimulation: some AAV injections into the TMN area of *HDC-Cre* mice missed the target, and so there was no ChR-YFP expression detected by primary fluorescence when we sliced the brains. Slices from these mice provided negative controls for light stimulation in the neocortex and caudate-putamen: the steady-state holding current recorded from layer IV cortical neurons with no ChR expressed in the TMN was -320 ± 140 pA before and -300 ± 130 pA after 5Hz light stimulation, demonstrating no significant change (reduction of -0.24 ± 0.13 nS; n = 7) in G_{tonic} (paired t-test, p = 0.1). For caudate-putamen slices with no *HDC-ChR-YFP* expression, on average there was also a 0.3 ± 0.1 nS (n = 4) decrease in G_{tonic} during the 5 Hz light stimulation.
