# Supplementary material for: Wakefulness Is Governed by GABA and Histamine Cotransmission
Source: Neuron. 2015 Jul 1;87(1):164–78. doi: 10.1016/j.neuron.2015.06.003 (PMC4509551; doi:10.1016/j.neuron.2015.06.003)
Supplement: Document S2. Article plus Supplemental Information [file mmc2.pdf]

Article

Neuron

Wakefulness Is Governed by GABA and Histamine Cotransmission

Highlights

- Histaminergic axons corelease GABA into the neocortex and striatum
- The released GABA produces slow tonic inhibition
- Reducing *vgat* expression in histaminergic neurons increases wakefulness
- Histamine-GABA axons will coordinate tonic inhibition over large cortical areas

Authors

Xiao Yu, Zhiwen Ye,
Catriona M. Houston, ...,
Nicholas P. Franks,
Stephen G. Brickley, William Wisden

Correspondence

n.franks@imperial.ac.uk (N.P.F.),
s.brickley@imperial.ac.uk (S.G.B.),
w.wisden@imperial.ac.uk (W.W.)

In Brief

Hypothalamic histamine neurons are well known as excitatory wake-promoting neurons. But they also contain GABA. In this paper, Yu et al. functionally demonstrate that histaminergic axons in the neocortex corelease a slow paracrine GABA signal to suppress wakefulness.

Yu et al., 2015, Neuron *87*, 164–178
July 1, 2015 ©2015 The Authors
http://dx.doi.org/10.1016/j.neuron.2015.06.003

CellPress

Neuron
Article

Wakefulness Is Governed by GABA and Histamine Cotransmission

Xiao Yu,[1,5] Zhiwen Ye,[1,5] Catriona M. Houston,[1] Anna Y. Zecharia,[1] Ying Ma,[1] Zhe Zhang,[1] David S. Uygun,[1] Susan Parker,[2] Alexei L. Vyssotski,[4] Raquel Yustos,[1] Nicholas P. Franks,[1,3,6,*] Stephen G. Brickley,[1,3,6,*] and William Wisden[1,3,6,*]
[1]Department of Life Sciences
[2]Department of Physics
[3]Centre for Neurotechnology
Imperial College London, London SW7 2AZ, UK
[4]Institute of Neuroinformatics, University of Zürich/ETH Zürich, Winterthurerstrasse 190, CH-8057, Zürich, Switzerland
[5]Co-first author
[6]Co-senior authors
*Correspondence: n.franks@imperial.ac.uk (N.P.F.), s.brickley@imperial.ac.uk (S.G.B.), w.wisden@imperial.ac.uk (W.W.)
http://dx.doi.org/10.1016/j.neuron.2015.06.003
This is an open access article under the CC BY license (http://creativecommons.org/licenses/by/4.0/).

SUMMARY

Histaminergic neurons in the tuberomammilary nucleus (TMN) of the hypothalamus form a widely projecting, wake-active network that sustains arousal. Yet most histaminergic neurons contain GABA. Selective siRNA knockdown of the vesicular GABA transporter (*vgat*, *SLC32A1*) in histaminergic neurons produced hyperactive mice with an exceptional amount of sustained wakefulness. Ablation of the *vgat* gene throughout the TMN further sharpened this phenotype. Optogenetic stimulation in the caudate-putamen and neocortex of "histaminergic" axonal projections from the TMN evoked tonic (extrasynaptic) GABA$_A$ receptor Cl$^-$ currents onto medium spiny neurons and pyramidal neurons. These currents were abolished following *vgat* gene removal from the TMN area. Thus wake-active histaminergic neurons generate a paracrine GABAergic signal that serves to provide a brake on overactivation from histamine, but could also increase the precision of neocortical processing. The long range of histamine-GABA axonal projections suggests that extrasynaptic inhibition will be coordinated over large neocortical and striatal areas.

INTRODUCTION

Histaminergic neurons in the tuberomammilary nucleus (TMN), a region of the posterior hypothalamus, help sustain wakefulness (Anaclet et al., 2009; Haas et al., 2008; Lin et al., 1988, 1989; Monnier et al., 1967; Nicholson et al., 1991; Parmentier et al., 2002, 2009; Saper et al., 2010; Wada et al., 1991; Yu et al., 2014). They are the sole source of neuronal histamine and send axons throughout the brain (Bayliss et al., 1990; Panula et al., 1984; Watanabe et al., 1983). TMN neurons become active just after waking and fire at an average rate of about 5 Hz, and their activity is suppressed during sleep (Sakai et al., 2010; Saper et al., 2010; Takahashi et al., 2006). Histamine modulates diverse circuitries (Ellender et al., 2011; Haas et al., 2008; Wada et al., 1991). Histamine can suppress, for example, glutamate or GABA inputs to local circuits by activating hetero-H3 metabotropic receptors on terminals; it can also depolarize cells via H1 and H2 metabotropic receptors or cause phosphorylation of ion channels that influence firing rate (Atzori et al., 2000; Ellender et al., 2011). These effects can occur in the same local circuitry. At the behavioral level, the net effects of histamine's actions on circuits are enhanced aspects of wakefulness such as cognition, locomotion, feeding, and motivation (Torrealba et al., 2012).

Physiological investigations have focused on the histaminergic aspect of TMN neurons. But these neurons also contain glutamic acid decarboxylase (GAD) enzymes and GABA itself (Airaksinen et al., 1992; Senba et al., 1985; Takeda et al., 1984; Trottier et al., 2002; Vincent et al., 1983). GABA's presence in histaminergic neurons is conserved, from fish through to humans, suggesting a core function (Sundvik and Panula, 2012; Trottier et al., 2002). It is not known what this function is, and there has been no demonstration that these cells actually release GABA. Optogenetic stimulation of histaminergic fibers in the ventral lateral preoptic (VLPO) area of the hypothalamus found that histamine release stimulated local GABAergic interneurons (Williams et al., 2014), but provided no evidence of GABA release from the histamine fibers.

Varicosities on TMN axons broadcast histamine by volume transmission—histaminergic neurons rarely use synapses (Takagi et al., 1986). So it is likely that if these same TMN axons do release GABA, then this particular source of GABA would also function, similar to histamine, in a paracrine manner that would principally affect extrasynaptic receptors. Because GABA is an electroneutral *zwitterion* at physiological pH, it has diminished interactions with the extracellular matrix. Thus GABA is well suited for diffusion over long distances (Roberts and Sherman, 1993). Ambient (nonsynaptic) GABA produces sustained inhibitory currents by activating high-affinity

CellPress

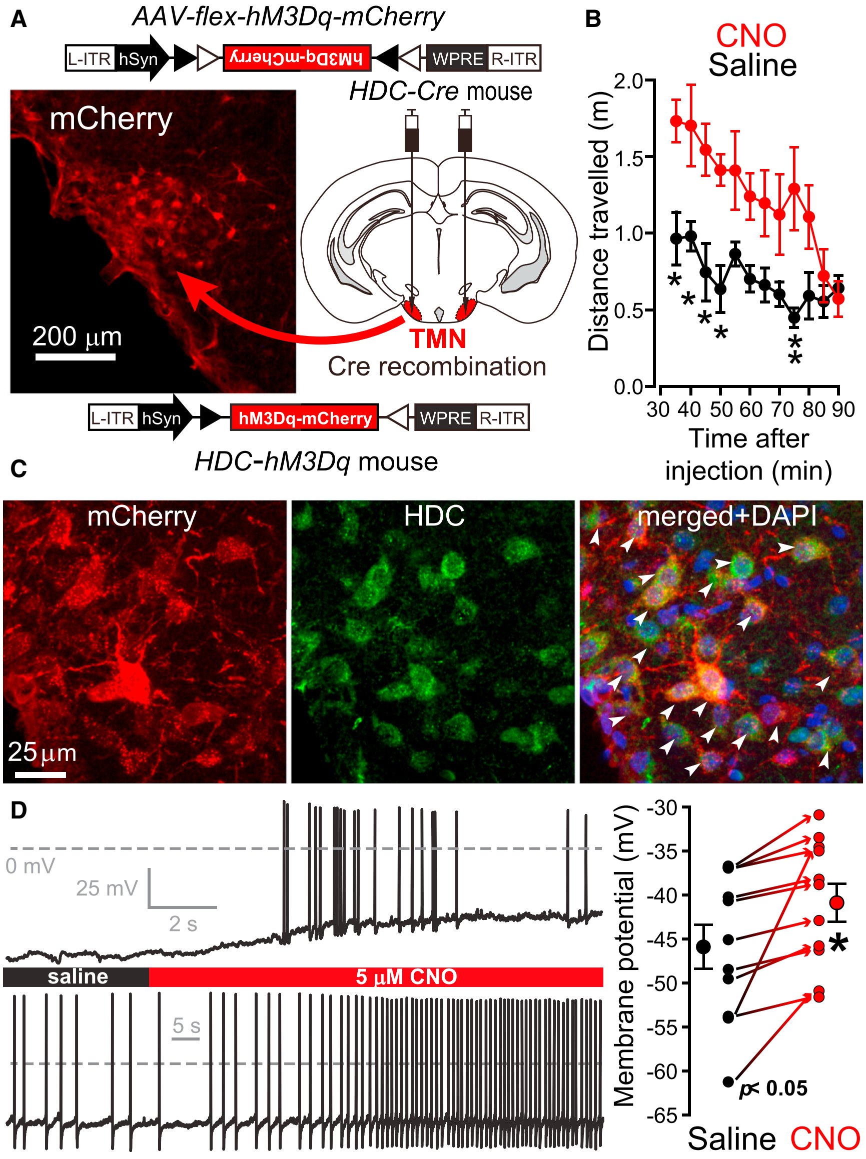

Figure 1. Pharmacogenetic Activation of Histaminergic Neurons Increased Motor Activity of *HDC-hM3Dq* Mice

(A) *AAV-flex-hM3Dq-mCherry* was bilaterally injected into the TMN region of *HDC-Cre* mice to give hM3Dq-mCherry expression selectively within histaminergic neurons (see inset image).

(B) Activity of *HDC-hM3Dq* mice in an open field arena 30 min after saline (black trace, n = 4 mice) or CNO (red trace, n = 4 mice) injection. The distance moved was calculated every 5 min and average values (mean ± SEM) plotted over 60 min (*p < 0.05; **p < 0.01).

(C) Double-label immunocytochemisty of HDC- and hM3Dq-mCherry-positive neurons with HDC antisera and mCherry antibody; arrowheads indicate examples of double-labeled neurons. DAPI labeling was included to locate all cells.

(D) Two examples of voltage traces recorded from *HDC-hM3Dq* mice during the application of CNO. The top trace shows an example of a silent neuron that was sufficiently depolarized to fire APs. The bottom trace shows a spontaneously active neuron with increased AP firing in the presence of CNO. On average there was a significant (paired t test, $p < 0.05$) ~5 mV depolarization of TMN neurons (n = 11 cells, control, −46 ± 3 mV; CNO, −41 ± 2 mV). The results from each cell are shown on the scatterplot on the right.

extrasynaptic ionotropic $GABA_A$ receptors (Brickley et al., 1996; Brickley and Mody, 2012; Lee and Maguire, 2014); this is termed the tonic inhibitory conductance (G_{tonic}).

In this paper we start by showing that selective pharmacogenetic stimulation of histamine neurons enhances behavioral arousal. We then demonstrate that knocking down or removing *vgat* gene expression from histaminergic neurons increases locomotion, and causes a substantial increase in wakefulness during the night. Optogenetically stimulating histaminergic fibers in the neocortex and caudate-putamen increases G_{tonic} onto pyramidal neurons and medium spiny cells, respectively. Eliminating *vgat* from histaminergic neurons selectively abolishes these evoked tonic inhibitory currents. Thus, during wakefulness, GABA can be deposited widely in neocortical and striatal circuitry by volume transmission. The GABA and histamine TMN components work together to regulate the amount of wakefulness.

RESULTS

Histaminergic Neurons Stimulate Arousal but Are Also GABAergic

We first tested the excitatory nature of histaminergic neurons on behavior by selectively stimulating them pharmacogenetically in vivo with DREADD hM3Dq-mCherry receptors and clozapine-N-oxide (CNO) (Alexander et al., 2009; Krashes et al., 2011). The *hM3Dq* receptor is an excitatory metabotropic receptor. Histaminergic neurons can be genetically targeted because of their unique expression of the histidine decarboxylase (*hdc*) gene, allowing the generation of *HDC-Cre* mice (Yu et al., 2014; Zecharia et al., 2012). The TMN area of *HDC-Cre* mice was bilaterally transduced with *AAV-flex-hM3Dq-mCherry* (Figure 1A) to produce *HDC-hM3Dq* mice. Intraperitoneal administration of CNO, but not saline, to these *HDC-hM3Dq* mice significantly increased their motor activity in an open area (Figure 1B) (saline, n = 4; CNO, n = 4, two-way ANOVA and post hoc Bonferroni, *p < 0.05; **p < 0.01; measurements taken 30 min post CNO or saline injection). In these mice, the *hM3Dq-mCherry* receptor was selectively expressed in most histaminergic neurons (348 mCherry-positive cells out of 405 HDC-positive cells were counted across three animals; 85% ± 3.5% of the HDC-neurons were mCherry positive) (Figure 1C). Using whole-cell recording in acute slices from the TMN area of *HDC-hM3Dq* mice (n = 4 mice), we demonstrated that CNO significantly (paired t test, $p < 0.05$) depolarized

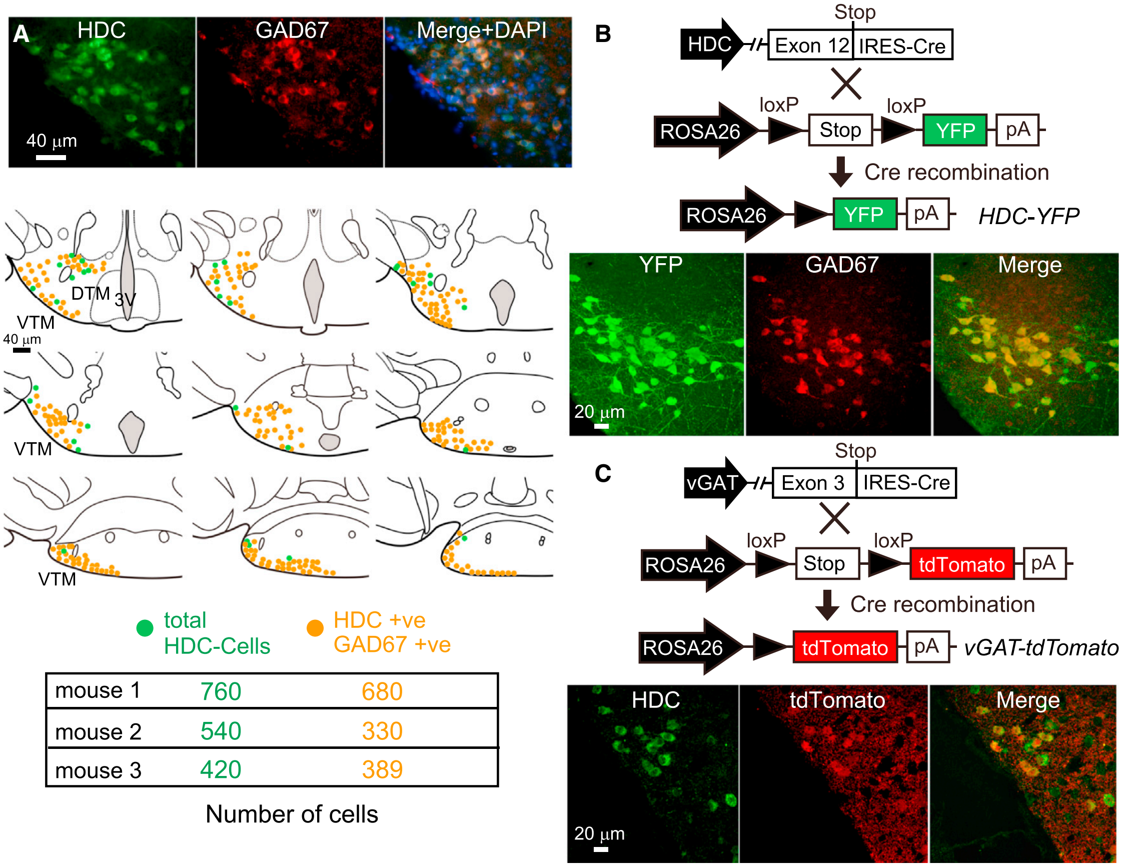

Figure 2. The Majority of Histaminergic Neurons Express GABAergic Markers

(A) Coronal section of the mouse posterior hypothalamus stained with HDC (green) and GAD67 (red) antisera. The diagram summarizes the staining from the whole TMN region: HDC-positive cells (green) and double-positive cells (orange). 3V, third ventricle; VTM, ventral tuberomammillary; DTM, diffuse tuberomammillary.

(B) *HDC-Cre* mice were crossed with *Rosa26-loxP-stop-loxP-YFP* mice to generate *HDC-YFP* mice. TMN sections from *HDC-YFP* mice were costained with EYFP and GAD67 antisera. Most YFP-positive (HDC neurons) in the TMN were also GAD67-positive.

(C) *vGAT-Cre* mice were crossed with *Rosa26-loxP-stop-loxP-tdTomato* mice to generate *vGAT-tdTomato* mice. Sections from *vGAT-tdTomato* mice were stained with HDC antisera. Most HDC neurons were tdTomato positive.

TMN neurons by ~5 mV (n = 11 cells, control, −46 ± 3 mV; CNO, −41 ± 2 mV). This was sufficient to elicit action potential (AP) firing in silent cells (5 out of 11) or increase AP firing rate in spontaneously active cells (Figure 1D). Thus, selectively activating histaminergic neurons generated hyperlocomotion and behavioral arousal.

In spite of this excitatory effect on behavior, histaminergic TMN cells contain GABA and its synthetic enzyme GAD67 (*gad1*) (Airaksinen et al., 1992; Takeda et al., 1984; Vincent et al., 1983). By immunocytochemistry, we found that a majority of HDC-positive neurons costained strongly with GAD67 antisera (1,399 GAD67-positive cells out of 1,720 HDC-positive cells were counted across three animals; 81% ± 10% of the HDC neurons were GAD67 positive; Figure 2A). We corroborated this result genetically by crossing *HDC-Cre* mice with conditional *rosa-lox-stop-lox-YFP* mice (Srinivas et al., 2001). In these crosses, 85% ± 5% (n = 3 mice) of YFP-positive neurons in the TMN also expressed GAD67 (Figure 2B). Conventionally, if histaminergic neurons were to release GABA, they should also express the vesicular GABA transporter (*vgat*, *Slc32a1*) gene. With the exception of midbrain dopamine neurons (Tritsch et al., 2012, 2014), vGAT has so far proved essential for all neurons that release GABA (Tong et al., 2008; Wojcik et al., 2006); *vgat* expression, however, has not been demonstrated for HDC cells. Crossing *vGAT-Cre* mice (Vong et al., 2011) with those containing a *rosa-lox-stop-lox-tdTomato* allele (Madisen et al., 2010) gave extensive tdTomato/HDC soma costaining in the TMN (Figure 2C). Thus histaminergic neurons express the *vgat* gene and have the potential to package GABA into vesicles.

Genetic Disruption of GABA Function in Histaminergic Neurons

To remove the putative GABAergic function from histaminergic neurons, we considered crossing either $vgat^{lox/lox}$ mice or double $gad1^{lox/lox}/gad2^{lox/lox}$ mice with the *HDC-Cre* mice. However, the *hdc-cre*, *vgat*, *gad1*, and *gad2* genes are all in the same region of mouse chromosome 2, which precluded easy generation of homozygous $HDC\text{-}Cre/vgat^{lox/lox}$ or $HDC\text{-}Cre/gad1^{lox/lox}/gad2^{lox/lox}$

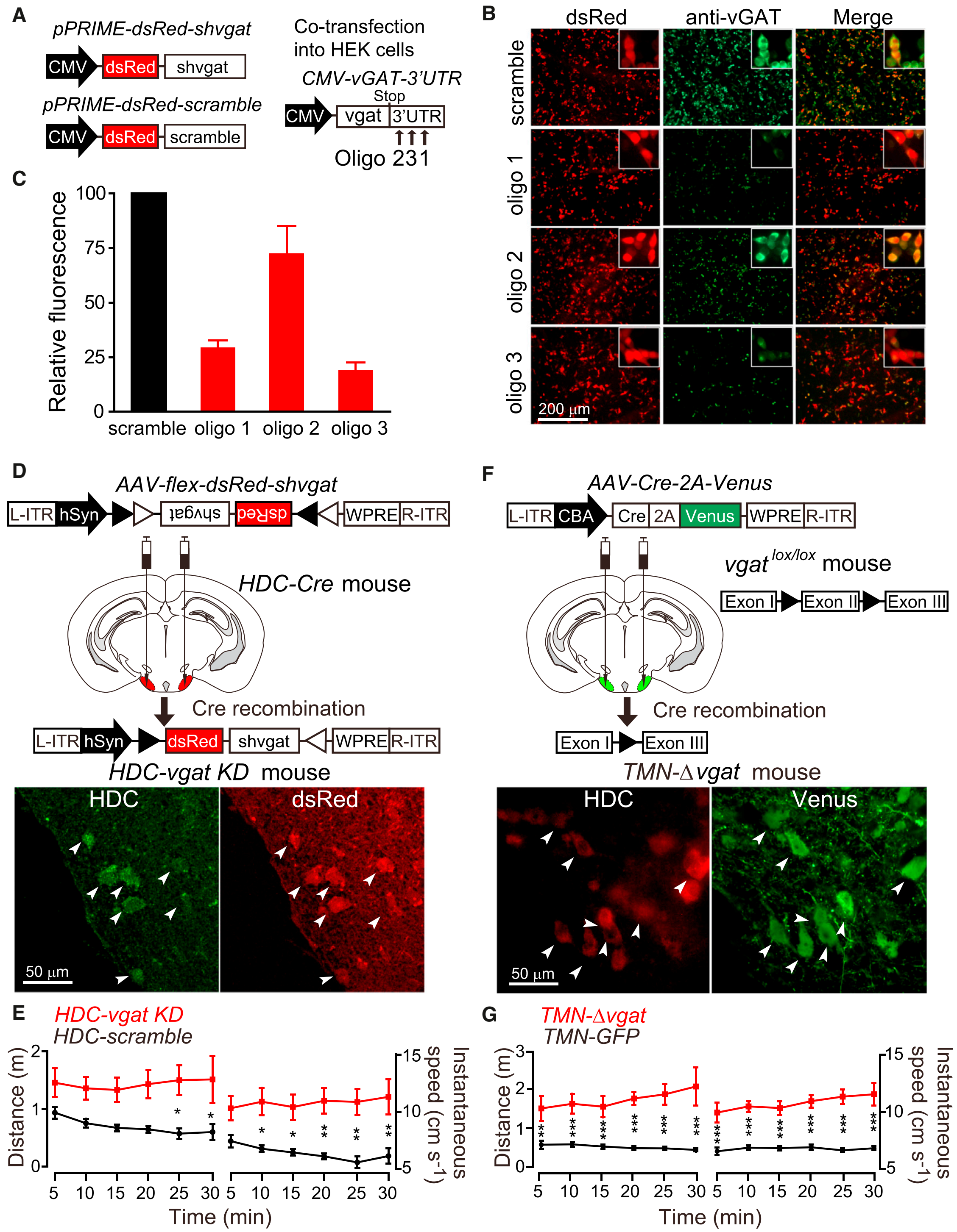

Figure 3. Knocking Down and Knocking Out *vgat* Expression from Histaminergic Neurons in the TMN

(A) Three hairpin (*sh*) oligonucleotides (*sholigo*), targeting the *vgat* transcript, were designed. Each *sholigo* was separately cloned into the *pPRIME-dsRed* vector; a scramble shRNA was also cloned into *pPRIME-dsRed*.

(B) To test the knockdown efficiency of the three *shvgat* oligonucleotides in vitro, *CMV-dsRed-scramble* or *CMV-dsRed-shvgat* (1, 2, or 3) were cotransfected with *pCMV-vGAT-3′UTR* (a cDNA containing the vgat open reading frame and 3′ untranslated region) into HEK293 cells. Thirty-six hours later, cells were fixed and stained with vGAT antisera. vGAT expression was best reduced with *sholigo1* and *sholigo3*.

(C) Three independent transfections were performed, and relative vGAT immunofluorescence in HEK cells was quantified.

(legend continued on next page)

animals. We therefore developed *vgat*-selective shRNA constructs to knock down *vgat* expression (Figure 3 and see Figure S1 available online). For this we placed putative *vgat* shRNA sequences into a microRNA gene, *mir30*, in the 3′ untranslated region of a transcript that also encodes dsRed (Stegmeier et al., 2005). These constructs were termed *dsRed-shvgat* (oligo1, oligo2, or oligo3) (Figure 3A). We tested how well these constructs could reduce *vgat* reporter gene expression in transfected HEK293 cells by cotransfecting *dsRed-shvgat* (oligo1, oligo2, or oligo3) with *CMV-vGAT*, respectively. Two shRNAs strongly knocked down *vgat* reporter gene expression in HEK cells (Figures 3B and 3C, $p < 0.05$, t test). Before moving to the more challenging area of the TMN, we used a striatum-based motor assay to demonstrate that the constructs, delivered in AAVs, worked effectively in vivo (Figure S1). To compare the behavior produced by knockdown and knockout of vGAT in the caudate-putamen (CPu), we unilaterally injected *AAV-dsRed-shvgat* and *AAV-Cre-2A-Venus* into wild-type and $vgat^{lox/lox}$ mice, respectively, to generate *CPu-vgat KD* and *CPu-Δvgat* mice (Figures S1A and S1D). Both *CPu-vgat KD* and *CPu-Δvgat* groups had significantly decreased *vgat* transcript levels (Figures S1B and S1E) and increased ipsilateral turning compared with controls (Figures S1C and S1F).

To knock down *vgat* expression selectively in histaminergic neurons, we placed the dsRed-shvgat and dsRed-scramble cassettes in reverse orientation between heterologous lox sites (a FLEX switch) (Figure 3D), so that expression could only be induced by CRE recombinase (Atasoy et al., 2008). The *AAV-flex-dsRed-shvgat* or *AAV-flex-dsRed-scramble* viruses were bilaterally injected into the TMN area of HDC-Cre mice (Figure 3D). The groups of mice are referred to as *HDC-vgat KD* and *HDC-scramble*, respectively. We also injected AAV-Cre-2A-Venus and *AAV-GFP* into the TMN area of $vgat^{lox/lox}$ mice to produce *TMN-Δvgat* and *TMN-GFP* mice, respectively (Figure 3F). In *TMN-Δvgat* mice, the injections covered the TMN area; 77% ± 2% of HDC-positive neurons expressed Cre-2A-Venus (Figure S2). In the TMN, all of the GAD67-positive neurons also contained HDC (Figure 2A). These *TMN-Δvgat* mice formed an approximate comparison for the *HDC-vgat KD* mice, with the caveat that some GABAergic cells in the TMN area in addition to the histaminergic cells could be affected. We tested the vgat gene expression levels in the TMN area of *HDC-vgat KD* and *TMN-Δvgat* mice by both qPCR on tissue punches and also single-cell qPCR from identified neurons in acute brain slices prepared from the TMN area (Figure S3). The tissue-punch analysis showed that in both *TMN-Δvgat* and *HDC-vgat KD* mice, vgat mRNA levels in the TMN were significantly decreased compared with those in *TMN-GFP* or *HDC-scramble* mice (*TMN-GFP*, 1 ± 0.06 versus *TMN-Δvgat*, 0.5 ± 0.06, t test, $^{**}p < 0.01$; *HDC-scramble*, 1 ± 0.04 versus *HDC-vgat KD*, 0.76 ± 0.05, t test, $^{*}p < 0.05$) (Figures S3A–S3C). The single-cell qPCR showed that in both *TMN-Δvgat* and *HDC-vgat KD* mice, the vgat mRNA levels from identified single neurons were also significantly decreased compared with those in *TMN-GFP* or *HDC-scramble* mice (*TMN-GFP*, 1 ± 0.46 versus *TMN-Δvgat*, 0.06 ± 0.02, t test, $^{*}p < 0.05$; *HDC-scramble*, 1 ± 0.24 versus *HDC-vgat KD*, 0.35 ± 0.17, t test, $^{*}p < 0.05$) (Figures S3D–S3G). The content of histamine in the neocortex and caudate-putamen of *HDC-vgat KD* and *TMN-Δvgat* mice was unchanged (see Figure S4).

HDC-vgat KD and *TMN-Δvgat* Mice Are Hyperactive

We first quantified the general activity of the mice. In an open field assay, *HDC-vgat KD* mice ran significantly further and had higher instantaneous speeds than the *HDC-scramble* controls, maintaining a stable and high activity for the 30 min duration of the test (Figure 3E; *HDC-scramble*, n = 7; *HDC-vgat KD*, n = 8, two-way ANOVA and post hoc Bonferroni, $^{*}p < 0.05$; $^{**}p < 0.01$). Similarly, the *TMN-Δvgat* mice were continuously more active compared with *TMN-GFP* control mice (Figure 3G; *TMN-GFP*, n = 10; *TMN-Δvgat*, n = 6, two-way ANOVA and post hoc Bonferroni, $^{**}p < 0.01$; $^{***}p < 0.001$). In fact, the activity of the *TMN-Δvgat* mice, as measured by instantaneous speed and distance covered, tended to increase toward the end of the experiment (Figure 3G).

HDC-vgat KD and *TMN-Δvgat* Mice Have Increased and Sustained Wakefulness during the Night

We used EEG/EMG analysis to assess the sleep-wake cycle (Figures 4 and S5A). The *HDC-vgat KD* and *TMN-Δvgat* mice exhibited similar changes in their sleep-wake behavior, largely confined to the night. First we describe the *HDC-vgat KD* (n = 6) and *HDC-scramble* (n = 6) groups (Figures 4A–4C and S5B). For the first half of the night, *HDC-vgat KD* mice had significantly more wake than the *HDC-scramble* controls (Figures 4A, 4B, and S5B) (wake, *HDC-scramble*, 400 ± 13 min versus *HDC-vgat KD*, 511 ± 15 min, t test, $^{***}p < 0.001$). The example in Figure 4A shows the EEG from an *HDC-vgat KD* mouse that was awake continuously for 6 hr during the first half of the night. In fact, during the night, *HDC-vgat KD* mice had only about 65% of normal NREM sleep compared with HDC-scramble mice (NREM, *HDC-scramble*, 293 ± 12 min versus *HDC-vgat KD*, 190 ± 14 min, t test, $^{***}p < 0.001$). Their REM sleep was also slightly decreased (*HDC-scramble*, 22 ± 2 min versus *HDC-vgat KD*, 17 ± 2 min, t test, $^{*}p < 0.05$). The EEG θ power of *HDC-vgat KD* mice was significantly increased selectively during the night (Figure 4C) (two-way ANOVA and post hoc Bonferroni, $^{***}p < 0.001$), consistent with the heightened wakefulness of

(D) *AAV-flex-dsRed-shvgat (oligo3)* was bilaterally injected into the TMN region of *HDC-Cre* mice. Cre recombination produced *dsRed-shvgat* expression in HDC-positive neurons, and the resulting mice were termed *HDC-vgat KD* mice. Arrowheads indicate examples of colabeled cells.

(E) *HDC-vgat KD* mice were more active than *HDC-scramble* (*AAV-flex-dsRed-scramble*-injected *HDC-Cre*) mice in an open field assay ($^{*}p < 0.05$; $^{**}p < 0.01$).

(F) *AAV-Cre-2A-Venus* was bilaterally injected into the TMN region of $vgat^{lox/lox}$ mice to generate *TMN-Δvgat* mice. Sections from virus-injected (*TMN-Δvgat*) mice were costained with HDC and GFP (Venus) antisera. In the TMN region, GFP expression was in 77% ± 2% of HDC neurons. Arrowheads indicate examples of colabeled cells.

(G) *TMN-Δvgat* mice ran further than *TMN-GFP* (*AAV-GFP*-injected $vgat^{lox/lox}$) mice in an open field assay ($^{**}p < 0.01$; $^{***}p < 0.001$). During each running episode *TMN-Δvgat* mice also ran faster, as evidenced by instantaneous speed measurements ($^{**}p < 0.01$; $^{***}p < 0.001$).

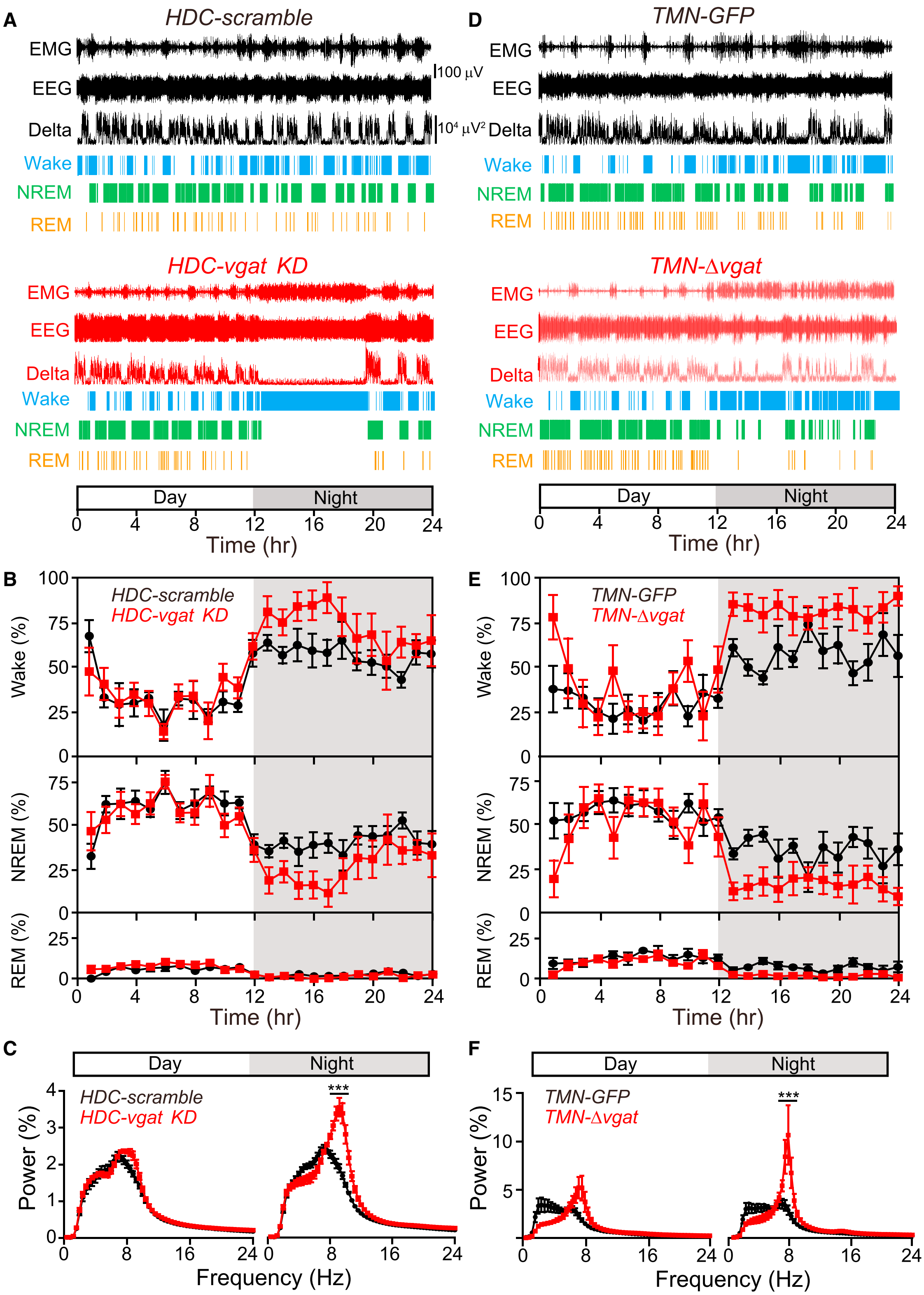

Figure 4. GABA Release from Histaminergic Neurons Governs the Amount of Sleep

(A) Continuous EMG, EEG, delta power, wake, NREM sleep, and REM sleep scoring data recorded for an *HDC-scramble* and an *HDC-vgat KD* mouse for 24 hr.

(B) The graphs illustrate the average 24 hr sleep scoring (percentage of wake, NREM, or REM sleep) for *HDC-scramble* (black trace) and *HDC-vgat KD* (red trace) mice; bars, SEM.

(legend continued on next page)

HDC-vgat KD mice (Figure 4B). In the day, both the *HDC-vgat KD* and *HDC-scramble* mice showed a similar sleep-wake pattern—with the same amount of NREM sleep and wake (Figures 4A, 4B, and S5B; wake, *HDC-scramble* (n = 6), 243 ± 10 min versus *HDC-vgat KD* (n = 6), 252 ± 18 min, t test, $p > 0.05$; NREM, *HDC-scramble* (n = 6), 425 ± 10 min versus *HDC-vgat KD* (n = 6), 409 ± 12 min, t test, $p > 0.05$).

Similar to *HDC-vgat KD* mice, *TMN-Δvgat* mice had a strong "sustained wakefulness" phenotype confined to the night (Figures 4D, 4E, and S5C). Compared with the *TMN-GFP* controls (n = 5), *TMN-Δvgat* (n = 7) animals were awake nearly the whole night (*TMN-GFP*, 411 ± 9 min versus *TMN-Δvgat*, 586 ± 27 min, t test, ***$p < 0.001$). Conversely, these *TMN-Δvgat* mice exhibited little NREM sleep during the dark period (Figures 4E and S5C; *TMN-GFP*, 258 ± 12 min versus *TMN-Δvgat*, 118 ± 25 min, t test, **$p < 0.01$). Their amount of REM sleep was also decreased (*TMN-GFP*, 48 ± 9 min versus *TMN-Δvgat*, 13 ± 3 min, t test, **$p < 0.01$). Similar to the *HDC-vgat KD* mice, selectively during the night, *TMN-Δvgat* mice had a strong increase of their maximum θ power frequency (Figure 4F). Thus both the *HDC-vgat KD* and *TMN-Δvgat* mice slept less. Their loss of sleep during the dark phase was not balanced by a corresponding increase in sleep duration during the day (two-way ANOVA and post hoc Bonferroni, ***$p < 0.001$).

After Sleep Deprivation, *HDC-vgat KD* and *TMN-Δvgat* Mice Still Sleep Less Than Control Mice, Maintaining Their Hyperactive State

Given the lack of sleep displayed by the *HDC-vgat KD* and *TMN-Δvgat* mice during a typical 24 hr cycle, we investigated how they behaved after sleep deprivation. Compared with control mice, would they demonstrate more recovery sleep to compensate for general lack of sleep during the normal sleep-wake cycle? Mice were sleep deprived for 5 hr during the beginning of the day by presenting them with novel objects. After sleep deprivation, mice were allowed recovery sleep in their home cages (Figure S6). Compared with the control mice, *HDC-vgat KD* mice had less NREM recovery sleep (Figures S6A and S6B), but the rate at which they reaccumulated their lost NREM sleep (~12.5 extra minutes NREM sleep recovered/hour) was similar to controls (Figure S6C). *TMN-Δvgat* mice behaved more extremely (Figures S6D and S6E). After sleep deprivation they were awake longer than control sleep-deprived mice and some 16 hr after sleep deprivation were spending most of their time awake (Figure S6E). However, as for the *TMN-Δvgat* mice, the rate at which they reaccumulated their lost NREM sleep (~12 extra minutes NREM sleep recovered/hour) was similar to controls (Figure S6F). Thus *HDC-vgat KD* and *TMN-Δvgat* mice sleep less than control mice, maintaining their hyperactive state even after sleep deprivation.

Histaminergic Axons in Neocortex and Striatum Release Paracrine GABA

Histamine neurons can globally coordinate behavioral states because their axons spread and diverge throughout the brain (Panula et al., 1989; Takagi et al., 1986; Wada et al., 1991). Can the axonal projections of histaminergic neurons release GABA, and does this release require vGAT? The behavioral results with *HDC-vgat KD* and *TMN-Δvgat* mice imply that this is the case. We addressed this directly using optogenetic stimulation in two types of mice: *HDC-Channel-Rhodopsin*(*ChR*) mice and *TMN-Δvgat/HDC-ChR* mice. To make *HDC-ChR* mice, the TMN area of *HDC-Cre* mice was bilaterally injected with *AAV-flex-ChR2H134R-EYFP* (Figures 5A and 5B). A TMN acute slice from *HDC-ChR* mice with ChR-EYFP primary fluorescence is shown in Figure 5B. The ChR-EYFP fusion protein was enriched in the membrane of HDC-neurons, including their axons (Figures 5B and 5C). Brief 1 ms stimuli were delivered to the LED every 200 ms, resulting in a 460 nm 0.1 mW/mm^2 peak light pulse that decayed with a time course of 10 msec. A continuous 5 Hz protocol was delivered for 3 min to HDC-ChR-EYFP-positive neurons in acute TMN slices (Figure 5D). This ChR stimulation protocol was capable of entraining action potential firing at 5 Hz (Figure 5E), corresponding to wake-active firing rates of TMN neurons (Takahashi et al., 2006). The ChR-EYFP fusion protein is an excellent substrate for anterograde axonal transport (Petreanu et al., 2007; Tye and Deisseroth, 2012). EYFP-positive fibers, marking the presence of ChR, were present in both the caudate-putamen (Figures 5F and 5G) and all neocortical areas examined e.g., sensory, motor, and visual neocortex (Figures 5H and 5I). The *TMN-Δvgat/HDC-ChR* mice have light-sensitive HDC neurons with deleted *vgat* gene expression. To make these, we coinjected *AAV-Cre-2A-Venus* and *AAV-flex-ChR2-EYFP* bilaterally into the TMN area of $vgat^{lox/lox}$ mice (Figures 6A and 6B).

We carried out functional circuit mapping by light-stimulating the ChR-EYFP labeled fibers in the neocortex (Figure 6C) and caudate-putamen (Figure 6J) prepared from *TMN-Δvgat/HDC-ChR* and *HDC-ChR* mice. Stimulation of *HDC-ChR* neocortical slices with brief light pulses delivered at 5 Hz generated slow and sustained increases in the holding current recorded, in whole-cell mode, from pyramidal cells (Figure 6D). This current resembled the G_{tonic} produced by activation of extrasynaptic $GABA_A$ receptors (Brickley et al., 1996; Brickley and Mody, 2012; Bright et al., 2007; Wisden et al., 2002). On average, G_{tonic} significantly increased by 1.1 ± 0.3 nS (paired t test, $p < 0.01$; n = 8) in the *HDC-ChR* slices (Figure 6E). In the example shown (Figure 6D), the enhancement of G_{tonic} was apparent after a few minutes of 5 Hz continuous optical stimulation, consistent with GABA diffusing to its target receptors from distant release sites (i.e., a paracrine action). To determine whether the GABA was

(C) Comparison of the power spectra of wake obtained from EEG data recorded during the day and night for the *HDC-scramble* (black trace) and *HDC-vgat KD* (red trace) mice.
(D) Continuous EMG, EEG, delta power, wake, NREM sleep, and REM sleep scoring data recorded from *TMN-GFP* and *TMN-Δvgat* mice.
(E) The graphs illustrate the average 24 hr sleep scoring (percentage of wake, NREM, or REM sleep) for *TMN-GFP* (black trace) and *TMN-Δvgat* (red trace) mice; bars, SEM.
(F) Comparison of the power spectra of wake obtained from EEG data recorded during the day and night for the *TMN-GFP* (black trace) and *TMN-Δvgat* (red trace) mice.

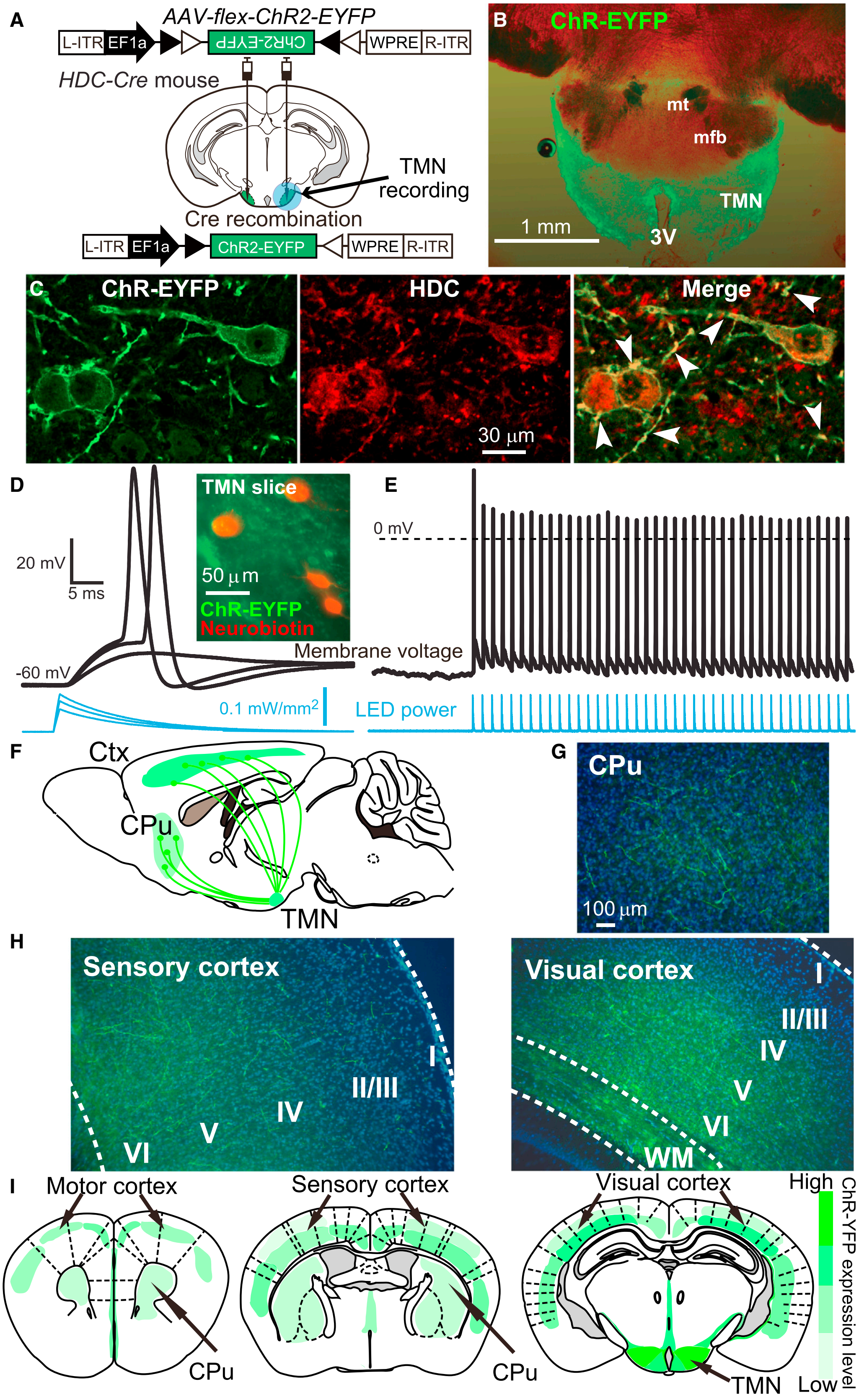

(legend on next page)

being released directly from HDC terminals or if this required an interneuron intermediate, we repeated these experiments in the presence of TTX (1 μM) and 4-AP (100 μM) (Root et al., 2014). A robust increase in G_{tonic} was observed, and on average it significantly increased by 0.5 ± 0.1 nS (paired t test, $p < 0.01$; n = 9) in the HDC-ChR slices (Figure 6F). In contrast, there was no increase in G_{tonic} in *TMN-Δvgat/HDC-ChR* slices following 5 Hz light stimulation (Figure 6G). On average G_{tonic} was slightly reduced by −0.2 ± 0.1 nS (n = 9) in the *TMN-Δvgat/HDC-ChR* mice (paired t test, p = 0.225, n = 9; Figure 6H). This suggests that vesicular GABA was being directly released from the histaminergic fibers using GABAergic (vGAT-dependent) vesicles to cause the increase in G_{tonic}. The results also imply that the G_{tonic} is not caused by activating any putative histamine-gated chloride channels (Fleck et al., 2012).

We next confirmed, independently of the HDC-specific *vgat* knockouts, that histamine release was not responsible for stimulating GABAergic interneurons to release GABA. We repeated the 5 Hz light stimulation of *HDC-ChR* fibers in the presence of the H1 receptor antagonist pyrilamine (20 μM) and the H2 receptor antagonist ranitidine (5 μM). A significant increase in G_{tonic} (0.6 ± 0.2 nS, n = 11; paired t test, $p < 0.05$) still occurred (Figure 6I), and as before, the increase was delayed after the onset of light stimulation. Applying H1/H2 receptor antagonists further suggested that there was no intermediary GABAergic interneuron involved that first required excitation by histamine, and which then released GABA to increase G_{tonic}. Instead, GABA release in the neocortex from TMN axons was vGAT dependent.

ChR-EYFP-positive fibers were also present in the caudate putamen of *HDC-ChR* mice (Figure 6J). During 5 Hz light stimulation, there was a gradual increase in G_{tonic} (1.9 ± 0.6 nS, n = 7; paired t test, $p < 0.05$) onto medium spiny neurons (Figure 6K). When the $GABA_A$ receptor antagonist gabazine (100 μM) was present during the stimulation, there was no increase in G_{tonic}; in fact, on average it decreased by −0.8 ± 0.5 nS (n = 6) (Figure 6L).

GABA Released from Histaminergic Axons Does Not Increase Phasic Inhibition, but Histamine Increases Synaptic Drive

We examined if GABA release from "GABA-histaminergic" axons in neocortex of *HDC-ChR* mice increased phasic (synaptic) $GABA_A$ receptor activation, in addition to its ability to raise G_{tonic}. The timing of sPSCs was analyzed relative to the LED trigger (Figure 7A). By cross-correlation analysis, sPSCs were not phase locked with ChR stimulation (Figure 7B) (n = 9 recordings from four *HDC-ChR* mice). However, the sPSC frequency significantly increased in these neocortical slices from 6.4 ± 1.7 Hz (n = 9) to 9.5 ± 2.2 Hz (paired t test, $p < 0.05$). This increase in asynchronous sPSC frequency was not observed when these experiments were repeated in the presence of TTX/4-AP (Figure 7C), although G_{tonic} did significantly increase (see Figure 6F). A similar increase in sPSC frequency was observed in the *TMN-Δvgat/HDC-ChR* mice, where the sPSC frequency increased from 8.7 ± 2.8 Hz (n = 9) to 15.6 ± 3.3 Hz (paired t test, $p < 0.05$). However, in these mice there was no increase in G_{tonic} (see Figure 6H). Does this light-evoked increase in sPSC rate depend on histamine release? In the presence of the H1 receptor antagonist pyrilamine (20 μM) and the H2 receptor antagonist ranitidine (5 μM), the ChR-stimulated increase in the rate of asynchronous sPSCs was blocked at the start and at the end of stimulation (Figure 7D; 23.9 ± 6.0 Hz [start] to 21.9 ± 5.4 Hz [end]; n = 11, paired t test, p = 0.3). For neocortical layer neurons, these data highlight the independent nature of the two signals from histaminergic-GABA axons originating from the TMN: an increased synaptic drive generated by histamine release and an increased G_{tonic} generated by GABA release.

DISCUSSION

Histamine neurons form a globally projecting hub that integrates brain functions associated with wakefulness (Wada et al., 1991). Starting in the TMN, histaminergic axons course large distances throughout the brain, generating extrasynaptic pools of transmitter. We showed that selective pharmacogenetic stimulation of these histamine neurons in vivo behaviorally excites the animals, as would be expected (Haas et al., 2008; Lin et al., 1988; Monnier et al., 1967; Nicholson et al., 1991). But we also established that histaminergic neurons use vGAT to release GABA. Similar to histamine, the GABA released from histaminergic neurons functions in a paracrine manner, which would make these neurons an unexpected source of GABAergic volume transmission in the neocortex and striatum. As part of their integrative role in specifying behavioral state, we expect that these hypothalamic TMN "GABA-histamine" neurons will contribute to tonic inhibition of many neural circuits simultaneously. Mice whose GABA-histamine neurons cannot release GABA are hyperactive, and they sleep less and do not catch up on this lost sleep. The

Figure 5. Selective ChR2-EYFP Expression in Histaminergic Neurons and Tracing Their Axons to Neocortex and Striatum

(A) *AAV-flex-ChR2-EYFP* was bilaterally injected into the TMN region of *HDC-Cre* mice. The blue circle indicates the area of the slice that was optically stimulated.

(B) Cre recombination produced *ChR2-EYFP* expression within HDC-expressing neurons. Shown is a combined bright-field and primary fluorescence photograph, taken at low magnification, of a freshly cut coronal brain slice used for electrophysiological recording from TMN neurons.

(C) *ChR-EYFP* expression in the TMN imaged with antisera to GFP (green) and HDC (red), with arrowheads indicating ChR2 expression in processes.

(D) The duration of the LED power output (blue trace) measured from the objective lens, and the membrane voltage recorded (black trace) from the soma of a *HDC-ChR2-EYFP* neuron. Increasing the LED power depolarized the membrane to generate action potentials. Inset image: four neurons recorded from this slice with cofluorescence for *ChR-EYFP* (green) and postrecording neurobiotin fill (red).

(E) The same cell as (D) firing action potentials with 5 Hz light stimulation.

(F) Schematic of the fibers (axons) which, following *AAV-flex-ChR2-EYFP* injection into the TMN of *HDC-Cre* mice, transported *ChR-EYFP* from the *HDC-ChR2-EYFP* soma into the neocortex and caudate-putamen.

(G and H) Low-power photographs of the *ChR2-EYFP*-positive fibers in the caudate-putamen (CPu) (G) and sensory and visual cortex (H). Blue, DAPI; I, layer I; II, layer II; III, layer III; IV, layer IV; V, layer V; VI, layer VI; WM, white matter.

(I) Schematic of the *ChR2-EYFP* fiber distribution in the caudate-putamen (CPu) and neocortex.

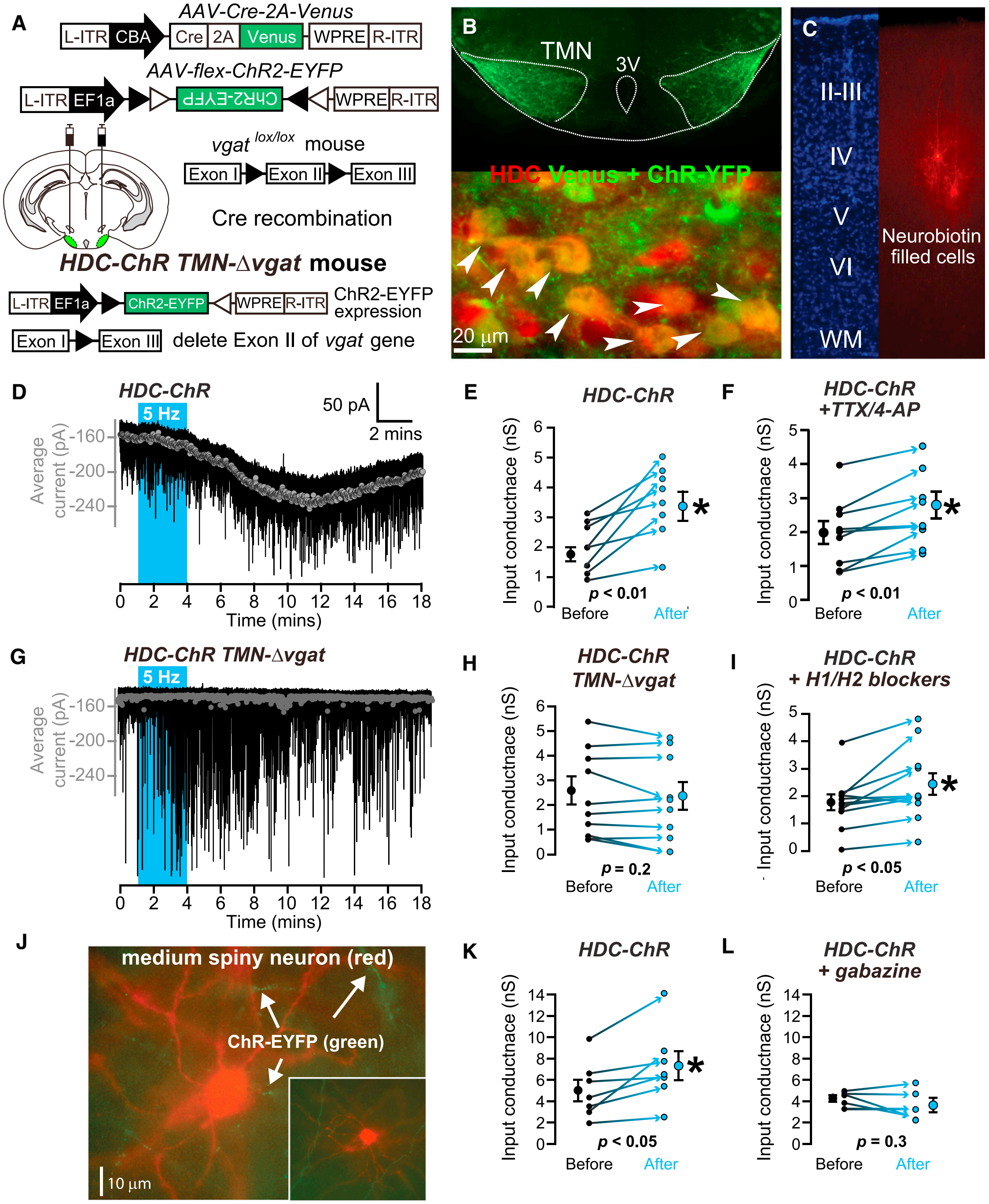

Figure 6. Histaminergic Axons Produce Slow GABAergic Responses in Visual Cortex Pyramidal and Striatal Neurons

(A) Double injection of *AAV-Cre-Venus* and *AAV-flex-ChR2-EYFP* into the TMN of *vgat*$^{lox/lox}$ mice to make light-sensitive histaminergic neurons that lack vGAT (*HDC-ChR TMN-Δvgat* mouse).

(B) The top photomicrograph shows combined expression of the two AAV transgenes in the bilateral TMN area (coronal section). The lower picture shows double staining in the TMN with HDC and EGFP antisera.

(C) Two layer IV pyramidal neurons filled with neurobiotin/Alexa 555 (red) postrecording.

(D) Electrophysiological data from a *HDC-ChR* mouse. The gray symbols superimposed upon the current trace (black line) show the average holding current calculated for every 1 s epoch during the entire recording. An increase in the holding current begins during the 5 Hz optogenetic stimulation but takes minutes to reach its peak response following termination of the stimuli.

(E) Scatterplot for all recordings made from the *HDC-ChR* mice. The lines indicate paired recordings made before and after the 5 Hz optogenetic stimulation. On average, G_{tonic} significantly increased by 1.1 ± 0.3 nS (paired t test, $p < 0.01$; n = 8) in the *HDC-ChR* slices.

(legend continued on next page)

"sleepless phenotype" manifests during the night. Their behavior might be described as a form of mania (Kirshenbaum et al., 2011), which in humans would be considered as part of a bipolar disorder.

Neurons often corelease small molecules and peptides (Adamantidis, 2015; Klausberger and Somogyi, 2008; Schöne and Burdakov, 2012; Tong et al., 2008; van den Pol, 2012). "Orexinergic" neurons, for example, in the lateral hypothalamus corelease glutamate and orexin onto histaminergic neurons in the TMN (Schöne et al., 2014). The different time course of the excitatory actions of the two transmitters (glutamate, fast synaptic excitation; orexin, slow and sustained excitation) allows them to signal different aspects of "metabolic integration" (Schöne et al., 2014). In addition to peptides, some neurons release several small molecule transmitters simultaneously, sometimes of the same functional type, e.g., GABA-glycine (Jonas et al., 1998), but often of functionally antagonist pairings, for example, GABA-glutamate, or GABA-acetylcholine or GABA-dopamine (Hnasko and Edwards, 2012; Vaaga et al., 2014). In contrast to our findings on GABA-histamine neurons, other investigations into GABA cotransmission or corelease, including cotransmission with peptides, found only fast monosynaptic responses generated by the GABA component (Jego et al., 2013; Root et al., 2014; Shabel et al., 2014; Tong et al., 2008; Tritsch et al., 2012). It might be expected that midbrain dopamine and histamine networks function similarly with respect to GABA release, but they do not. GABA is not synthesized in the dopamine neurons themselves—unlike the histamine neurons, they do not express the *gad1* or *gad2* genes—but GABA is instead scavenged into dopaminergic terminals by the transporters mGAT1 and mGAT4; it is hypothesized that GABA is then transported into dopamine vesicles by the monoamine transporter VMAT2 (Tritsch et al., 2012, 2014). For histamine neurons, *vgat* knockout removes GABA release; the VMAT2 in histamine neurons does not substitute for vGAT. In cultured TMN neurons from embryos, GABA is in putative vesicles distinct from histaminergic ones, suggesting cotransmission rather than corelease (Kukko-Lukjanov and Panula, 2003).

There is an intriguing potential for synergy directly at the receptor level between histamine and GABA. At (high) concentrations of around 1 mM, histamine is an effective positive allosteric modulator of α4 subunit-containing $GABA_A$ receptors (Bianchi et al., 2011), which are the type of $GABA_A$ receptors often associated with extrasynaptic activation (Brickley and Mody, 2012). However, when we removed *vgat* from histamine (HDC-ChR) axons, this abolished the light-evoked increase in tonic inhibition on layer IV pyramidal neurons, suggesting that the histamine released was not sufficient to activate any α4 subunit-containing $GABA_A$ receptors that might have been present. Similarly, the *vgat* deletion experiment also ruled out that the increase in G_{tonic} was due to activating (speculative) histamine-gated chloride channels (Fleck et al., 2012). In the absence of vGAT, light triggered a general increase in synaptic drive onto pyramidal neurons, which was blocked by H1 and H2 receptor antagonists. This effect of histamine is likely to reflect the engagement of multiple changes in the inputs impinging onto pyramidal neurons, as described for striatal neurons (Ellender et al., 2011). As the histamine system innervates most of the brain, further studies will be required to see if GABA is coreleased in other areas. In the ventral lateral preoptic hypothalamus, histamine axons do not release GABA when stimulated optogentically, but instead activate GABA interneurons by histamine release (Williams et al., 2014). By contrast, our experiments using TTX in the neocortex suggest that in this brain region no intermediary interneurons convey TMN axon-evoked GABA release. About 20% of histamine neurons in the TMN only contain histamine (Figure 2A), and these could be the ones that innervate VLPO.

The "GABA-histamine" TMN neurons only fire during wakefulness, so GABA will be released from their axons into the neocortex and striatum during the wake state. The paracrine nature of the GABA signal explains the lack of any phasic synaptic inhibition associated with GABA release from the histamine axons. Why would GABA-histamine neurons use contradictory "stop-go" signals? One reason could be to stop networks getting too excited by histamine: the *HDC-vgat KD* and *TMN-Δvgat* mice are more active and sleep less because the histaminergic system is overactive. The function of sleep is unknown, but an overactive histamine system, resulting in less sleep, may damage health and cause mania. GABA release from histamine neurons could keep the animal in the "optimal arousal zone."

A second reason could be related to the fact that in the awake neocortex, inhibition enhances processing (Haider et al., 2013). Indeed, the wake-active GABAergic volume transmission generated by TMN neurons may sharpen cognitive responses, and so GABA could work together with histamine to enhance wakefulness. Increased ambient GABA levels will generate greater G_{tonic}, which by speeding up the

(F) Scatterplot for all recordings made from the HDC-ChR mice in the presence of TTX and 4-AP. On average, G_{tonic} significantly increased by 0.5 ± 0.1 nS (paired t test, $p < 0.005$; n = 9) in the HDC-ChR slices.

(G) Electrophysiological data from a *HDC-ChR TMN-Δvgat* mouse. The gray symbols superimposed upon the current trace (black line) show the average holding current calculated every 1 s epoch for the entire recording. No change in the holding current occurred in response to the 5 Hz optogenetic stimulation, but there was an increase in the frequency of spontaneous synaptic activity (transient downward deflections).

(H) Scatterplots for recordings made from the *HDC-ChR TMN-Δvgat* mice. The lines indicate the paired recordings made before and after the 5 Hz optogenetic stimulation. G_{tonic} was reduced by −0.2 ± 0.1 nS in the *TMN-Δvgat/HDC-ChR* mice (paired t test, p = 0.225, n = 9).

(I) Scatterplots for recordings made from the *HDC-ChR* mice in the presence of H1 (pyrilamine) and H2 (ranitidine) blockers. G_{tonic} increased by 0.6 ± 0.2 nS (paired t test, $p < 0.05$; n = 11).

(J) Medium spiny neuron (red, neurobiotin fill postrecording) from the caudate-putamen of a *HDC-ChR* mouse; several *ChR-EYFP*-positive axons (green) are in the vicinity of the cell.

(K and L) Scatterplots for all recordings made from medium spiny neurons in the *HDC-ChR* mice in control conditions and with the $GABA_A$ receptor antagonist gabazine (10 μM). G_{tonic} increased by 1.9 ± 0.6 nS (paired t test, $p < 0.05$; n = 7) in control conditions and decreased by −0.8 ± 0.5 nS in the presence of gabazine (paired t test, p = 0.3; n = 6).

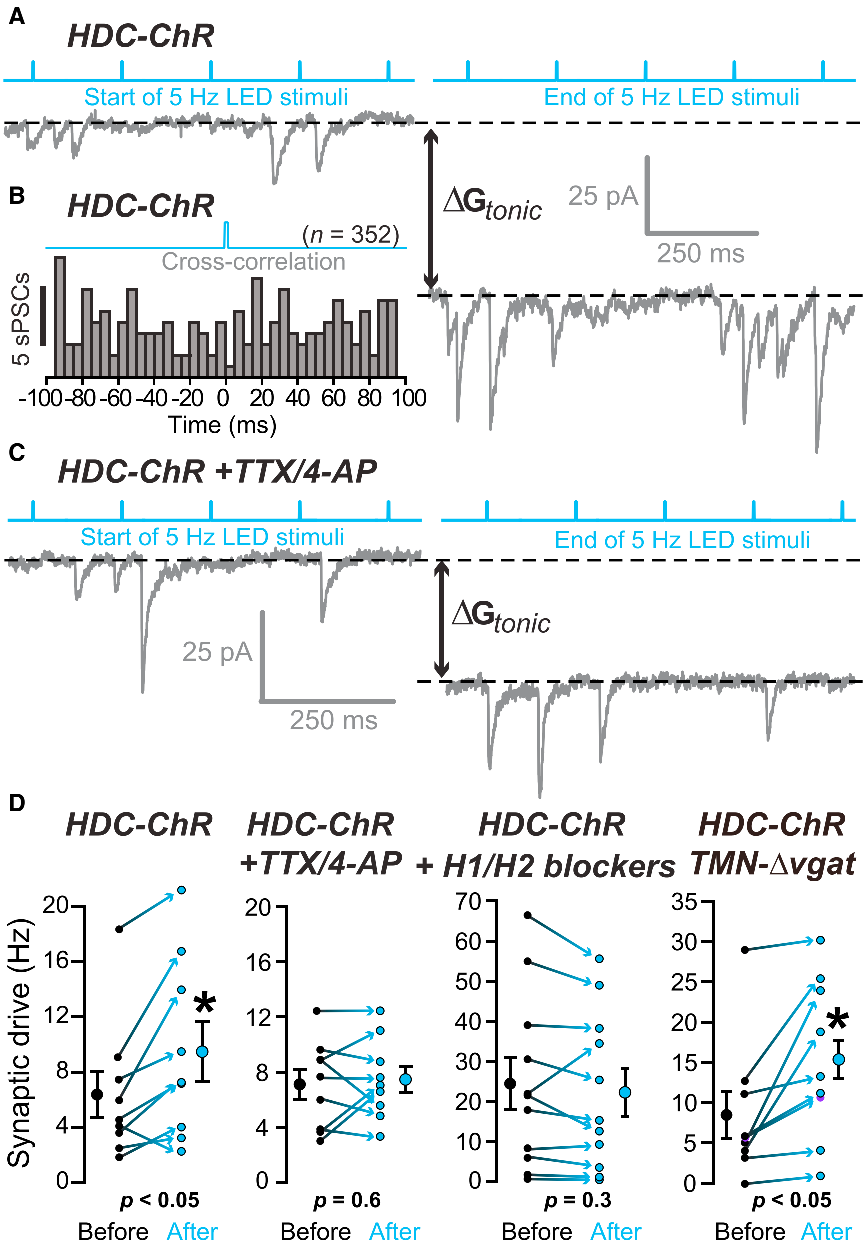

Figure 7. Activated Neocortical "GABA-Histamine" Axons Increase Synaptic Drive and G_{tonic} by Independent Mechanisms

(A) Whole-cell voltage-clamp recording from a pyramidal cell in layer IV of visual cortex during 5 Hz LED stimulation of axon fibers from an *HDC-ChR* mouse. The left-hand trace is from the start, and the right-hand trace is at the end of, the 5 Hz LED stimuli. The increased holding current between the first and last trace was used to calculate the increase in tonic conductance (ΔG_{tonic}).

(B) Cross-correlation analyses between the LED trigger and the occurrence of sPSCs. There was no peak in the histogram, consistent with a lack of LED-triggered PSCs.

(C) Whole-cell voltage-clamp recording in a layer IV pyramidal cell in the presence of TTX and 4-AP during 5-Hz LED stimulation of axon fibers in the visual neocortex from an *HDC-ChR* mouse. There was an increase in G_{tonic} but in this experiment little change in sPSC rate.

(D) Scatterplot of the change in synaptic drive (Hz) onto layer IV visual cortex pyramidal cells for the four main experiments: stimulation of *HDC-ChR* axons, stimulation of *HDC-ChR* axons in the presence of TTX/4-AP, stimulation of *HDC-ChR* axons in the presence of H1 and H2 receptor antagonists, and stimulation of *TMN-Δvgat/HDC-ChR* axons. In *HDC-ChR* mice the sPSC frequency significantly increased from 6.4 ± 1.7 Hz (n = 9) to 9.5 ± 2.2 Hz (paired t test, $p < 0.05$). In *TMN-Δvgat/HDC* mice, a similar increase in sPSC frequency was observed from 8.7 ± 2.8 Hz (n = 9) to 15.6 ± 3.3 Hz (paired t test, $p < 0.05$). With the H1 receptor antagonist pyrilamine (20 μM) and the H2 receptor antagonist ranitidine (5 μM), the ChR-stimulated increase in the rate of asynchronous sPSCs was 23.9 ± 6.0 Hz at the start and 21.9 ± 5.4 Hz at the end of stimulation (n = 11, paired t test, p = 0.3).

membrane time constant will increase the requirement for EPSP-spike precision, and constrain coincidence detection so that only closely timed inputs trigger action potentials (Brickley and Mody, 2012; Bright et al., 2007; Duguid et al., 2012; Hamann et al., 2002; Wlodarczyk et al., 2013). The long distances traversed by the GABA-histamine axons mean that extrasynaptic inhibition could be coordinated by the TMN over large cortical areas.

In conclusion, we think it is no longer correct to refer to TMN neurons as "histaminergic": the GABA arm of their activity is so striking that they might be referred to as "GABA-histamine" neurons. We have demonstrated that the wide-ranging GABA-histamine axons of the hypothalamic TMN broadcast dual inhibitory-excitatory signals in the neocortex: an increased G_{tonic} generated by GABA release, and an increased synaptic drive generated by histamine. The two components may work together to regulate the amount of wakefulness. If this balance of GABA-histamine cotransmission changes unfavorably, mania-like behaviors could emerge.

EXPERIMENTAL PROCEDURES

Animals

All experiments were performed in accordance with the UK Home Office Animal Procedures Act (1986); all procedures were approved by the Imperial College Ethical Review Committee. The following strains of mice were used: *HDC-Cre* (129SVJ and C57BL/6J) (Zecharia et al., 2012) (JAX stock number 021198); *Rosa26-loxP-Stop-loxP-YFP* (C57BL/6J), kindly provided by F. Costantini (Srinivas et al., 2001); $vgat^{lox/lox}$ (C57BL/6J and 129S4), kindly provided by B.B. Lowell (Tong et al., 2008) (Jax stock number 012897); *vGAT-Cre*, kindly provided by B.B. Lowell (Vong et al., 2011); and

Rosa26-loxP-Stop-loxP-tdTomato, kindly provided by H. Zeng (Madisen et al., 2010). See Supplemental Information for genotyping.

AAV Transgene Plasmids

Plasmid *pAAV-iCre-2A-Venus* was provided by Thomas Kuner (Abraham et al., 2010). Plasmid *pAAV-GFP* was a gift from John T. Gray (Addgene plasmid 32396). Plasmid *pAAV-EF1α-flex-ChR2H134R-EYFP* was a gift from Karl Deisseroth (Addgene plasmid 20298). The H134R mutation enhances light-evoked ChR currents and reduces spike-frequency adaptation (Nagel et al., 2005; Pulver et al., 2009). Plasmid *pAAV-hSyn-flex-hM3Dq-mCherry* was a gift from Bryan L. Roth (Addgene plasmid 44361) (Krashes et al., 2011). The *pPRIME* system, cloned into AAV transgenes, was used to generate shRNAs in vivo (Stegmeier et al., 2005). See Supplemental Information for shRNA sequences.

Adeno-Associated Virus Preparation and Stereotaxic Injections

To produce adeno-associated virus (AAV), the adenovirus helper plasmid *pFΔ6*, the AAV helper plasmids *pH21* (AAV1) and *pRVI* (AAV2), and the *pAAV* transgene plasmids were cotransfected into HEK293 cells and the subsequent AAV particles harvested on heparin columns, as described previously (Klugmann et al., 2005). To deliver the AAV into the brain, stereotaxic injections were performed using an Angle Two apparatus (Leica) linked to a digital brain atlas (Leica Biosystems Richmond, Inc.). Before injection, 1 μl AAV virus was mixed with 1 μl 20% mannitol (MERCK K93152782 111). The virus and mannitol mixture were injected into a pulled-glass pipette (Warner Instruments; OD = 1.00 mm, ID = 0.78 mm, length = 7.5 cm). Virus was injected at a speed of 25 nl/min. Virus (1 μl) was bilaterally injected into the brain, 0.5 μl for each side. For TMN, the injection coordinates were as follows: ML (−0.92 mm), AP (−2.70 mm), DV (−5.34 mm); ML (0.92 mm), AP (−2.70 mm), and DV (−5.34 mm). For unilateral injections, the injection coordinates were as follows: CPu, ML (−2.10 mm), AP (0.50 mm), and DV (−3.66 mm).

Histamine Measurements

Neocortex or striatum was collected and homogenized with 10 μl of 0.2 M perchloric acid per mg tissue and centrifuged at 10,000 rpm for 5 min at 4°C. The supernatants were collected and neutralized with an equal volume of 1 M potassium borate buffer. Brain histamine levels were determined with an ELISA kit (Beckman Coulter Co. number IM2562).

qPCR on Tissue Punches and Single-Cell qPCR

Total RNA from tissue punches was extracted using Trizol (Invitrogen). Reverse transcription and PCR were performed using a TaqMan RNA-to-Ct 1-Step Kit (Life Technologies, 4392653) and an Applied Biosystems machine (Foster City, USA). Acute slices of the TMN area were made (see "Electrophysiology"). AAV-transduced neurons were identified by fluorescence. After patching and recording (see "Electrophysiology"), we used the Single-Cell-to-CT Kit (Ambion). The content of the neuron was aspirated into the recording pipette and expelled into cell lysis/DNase I solution. Reverse transcription and cDNA preamplification were performed according to the kit protocol. qPCR was performed using the TaqMan Gene Expression Assay system. The TaqMan assay probes were designed and purchased from Invitrogen (UK): *mvgat*, Mm00494138_m1; *m18srRNA*, Mm03928990_g1; and *mhdc*, Mm00456104_m1.

EEG Analysis and Sleep-Wake Behavior

EEG and EMG signals were recorded using Neurologger 2A devices (Anisimov et al., 2014; Yu et al., 2014). The sleep state (wake, W; non-rapid eye movement, NREM; rapid eye movement, REM; doubt, D) was scored manually. See Supplemental Information for details. The sleep deprivation procedure, with presentation of novel objects, was performed as described (Yu et al., 2014).

General Behavioral Activity: Open Field Assay

All experiments were performed during "lights off" (active phase). The locomotion activity was detected in an activity test chamber with infrared sensors (Med Associates, Inc). The "instantaneous speed" was calculated as the distance traveled divided by the time spent moving (but not including the time the mice spent in the stationary state). For the DREADD experiments, clozapine-N-oxide (C0832, Sigma-Aldrich, dissolved in saline, 5 mg/kg) or saline was administered i.p. 30 min before the start of the behavioral observations.

Electrophysiology

Acute brain slices were prepared as described in Supplemental Information. For optogenetic experiments, a 470 nm collimated LED (M470L3-C1, Thorlabs) was used to illuminate the slice through the objective lens, and current-clamp recordings were first made from histaminergic neurons to confirm ChR2 expression. Subsequent voltage-clamp experiments in neocortex and stratum were used for cross-correlation analysis of synaptic events using a 100 ms sliding window before and after each LED pulse. Changes in the tonic $GABA_A$ receptor-meditated conductance (G_{tonic}) were calculated from the average holding current.

SUPPLEMENTAL INFORMATION

Supplemental Information includes six figures and Supplemental Experimental Procedures and can be found with this article at http://dx.doi.org/10.1016/j.neuron.2015.06.003.

ACKNOWLEDGMENTS

This work was supported by the Medical Research Council, UK (G0901892, S.G.B., N.P.F., and W.W.; G0800399, W.W.), the BBSRC (G021691 and BB/K018159/1, S.G.B., N.P.F., and W.W.), the Wellcome Trust (WT094211MA, S.G.B., N.P.F., and W.W.), and a UK-China Scholarships for Excellence/China Scholarship scheme (X.Y. and Z.Y.).

Received: January 9, 2015
Revised: April 29, 2015
Accepted: May 27, 2015
Published: June 18, 2015

REFERENCES

Abraham, N.M., Egger, V., Shimshek, D.R., Renden, R., Fukunaga, I., Sprengel, R., Seeburg, P.H., Klugmann, M., Margrie, T.W., Schaefer, A.T., and Kuner, T. (2010). Synaptic inhibition in the olfactory bulb accelerates odor discrimination in mice. Neuron *65*, 399–411.

Adamantidis, A.R. (2015). Sleep: the sound of a local alarm clock. Curr. Biol. *25*, 49–50.

Airaksinen, M.S., Alanen, S., Szabat, E., Visser, T.J., and Panula, P. (1992). Multiple neurotransmitters in the tuberomammillary nucleus: comparison of rat, mouse, and guinea pig. J. Comp. Neurol. *323*, 103–116.

Alexander, G.M., Rogan, S.C., Abbas, A.I., Armbruster, B.N., Pei, Y., Allen, J.A., Nonneman, R.J., Hartmann, J., Moy, S.S., Nicolelis, M.A., et al. (2009). Remote control of neuronal activity in transgenic mice expressing evolved G protein-coupled receptors. Neuron *63*, 27–39.

Anaclet, C., Parmentier, R., Ouk, K., Guidon, G., Buda, C., Sastre, J.P., Akaoka, H., Sergeeva, O.A., Yanagisawa, M., Ohtsu, H., et al. (2009). Orexin/hypocretin and histamine: distinct roles in the control of wakefulness demonstrated using knock-out mouse models. J. Neurosci. *29*, 14423–14438.

Anisimov, V.N., Herbst, J.A., Abramchuk, A.N., Latanov, A.V., Hahnloser, R.H., and Vyssotski, A.L. (2014). Reconstruction of vocal interactions in a group of small songbirds. Nat. Methods *11*, 1135–1137.

Atasoy, D., Aponte, Y., Su, H.H., and Sternson, S.M. (2008). A FLEX switch targets Channelrhodopsin-2 to multiple cell types for imaging and long-range circuit mapping. J. Neurosci. *28*, 7025–7030.

Atzori, M., Lau, D., Tansey, E.P., Chow, A., Ozaita, A., Rudy, B., and McBain, C.J. (2000). H2 histamine receptor-phosphorylation of Kv3.2 modulates interneuron fast spiking. Nat. Neurosci. *3*, 791–798.

Bayliss, D.A., Wang, Y.M., Zahnow, C.A., Joseph, D.R., and Millhorn, D.E. (1990). Localization of histidine decarboxylase mRNA in rat brain. Mol. Cell. Neurosci. *1*, 3–9.

Bianchi, M.T., Clark, A.G., and Fisher, J.L. (2011). The wake-promoting transmitter histamine preferentially enhances α-4 subunit-containing GABAA receptors. Neuropharmacology *61*, 747–752.

Brickley, S.G., and Mody, I. (2012). Extrasynaptic GABA(A) receptors: their function in the CNS and implications for disease. Neuron *73*, 23–34.

Brickley, S.G., Cull-Candy, S.G., and Farrant, M. (1996). Development of a tonic form of synaptic inhibition in rat cerebellar granule cells resulting from persistent activation of GABAA receptors. J. Physiol. *497*, 753–759.

Bright, D.P., Aller, M.I., and Brickley, S.G. (2007). Synaptic release generates a tonic GABA(A) receptor-mediated conductance that modulates burst precision in thalamic relay neurons. J. Neurosci. *27*, 2560–2569.

Duguid, I., Branco, T., London, M., Chadderton, P., and Häusser, M. (2012). Tonic inhibition enhances fidelity of sensory information transmission in the cerebellar cortex. J. Neurosci. *32*, 11132–11143.

Ellender, T.J., Huerta-Ocampo, I., Deisseroth, K., Capogna, M., and Bolam, J.P. (2011). Differential modulation of excitatory and inhibitory striatal synaptic transmission by histamine. J. Neurosci. *31*, 15340–15351.

Fleck, M.W., Thomson, J.L., and Hough, L.B. (2012). Histamine-gated ion channels in mammals? Biochem. Pharmacol. *83*, 1127–1135.

Haas, H.L., Sergeeva, O.A., and Selbach, O. (2008). Histamine in the nervous system. Physiol. Rev. *88*, 1183–1241.

Haider, B., Häusser, M., and Carandini, M. (2013). Inhibition dominates sensory responses in the awake cortex. Nature *493*, 97–100.

Hamann, M., Rossi, D.J., and Attwell, D. (2002). Tonic and spillover inhibition of granule cells control information flow through cerebellar cortex. Neuron *33*, 625–633.

Hnasko, T.S., and Edwards, R.H. (2012). Neurotransmitter corelease: mechanism and physiological role. Annu. Rev. Physiol. *74*, 225–243.

Jego, S., Glasgow, S.D., Herrera, C.G., Ekstrand, M., Reed, S.J., Boyce, R., Friedman, J., Burdakov, D., and Adamantidis, A.R. (2013). Optogenetic identification of a rapid eye movement sleep modulatory circuit in the hypothalamus. Nat. Neurosci. *16*, 1637–1643.

Jonas, P., Bischofberger, J., and Sandkühler, J. (1998). Corelease of two fast neurotransmitters at a central synapse. Science *281*, 419–424.

Kirshenbaum, G.S., Clapcote, S.J., Duffy, S., Burgess, C.R., Petersen, J., Jarowek, K.J., Yücel, Y.H., Cortez, M.A., Snead, O.C., 3rd, Vilsen, B., et al. (2011). Mania-like behavior induced by genetic dysfunction of the neuron-specific Na+,K+-ATPase α3 sodium pump. Proc. Natl. Acad. Sci. USA *108*, 18144–18149.

Klausberger, T., and Somogyi, P. (2008). Neuronal diversity and temporal dynamics: the unity of hippocampal circuit operations. Science *321*, 53–57.

Klugmann, M., Symes, C.W., Leichtlein, C.B., Klaussner, B.K., Dunning, J., Fong, D., Young, D., and During, M.J. (2005). AAV-mediated hippocampal expression of short and long Homer 1 proteins differentially affect cognition and seizure activity in adult rats. Mol. Cell. Neurosci. *28*, 347–360.

Krashes, M.J., Koda, S., Ye, C., Rogan, S.C., Adams, A.C., Cusher, D.S., Maratos-Flier, E., Roth, B.L., and Lowell, B.B. (2011). Rapid, reversible activation of AgRP neurons drives feeding behavior in mice. J. Clin. Invest. *121*, 1424–1428.

Kukko-Lukjanov, T.K., and Panula, P. (2003). Subcellular distribution of histamine, GABA and galanin in tuberomamillary neurons in vitro. J. Chem. Neuroanat. *25*, 279–292.

Lee, V., and Maguire, J. (2014). The impact of tonic GABAA receptor-mediated inhibition on neuronal excitability varies across brain region and cell type. Front. Neural Circuits *8*, 3.

Lin, J.S., Sakai, K., and Jouvet, M. (1988). Evidence for histaminergic arousal mechanisms in the hypothalamus of cat. Neuropharmacology *27*, 111–122.

Lin, J.S., Sakai, K., Vanni-Mercier, G., and Jouvet, M. (1989). A critical role of the posterior hypothalamus in the mechanisms of wakefulness determined by microinjection of muscimol in freely moving cats. Brain Res. *479*, 225–240.

Madisen, L., Zwingman, T.A., Sunkin, S.M., Oh, S.W., Zariwala, H.A., Gu, H., Ng, L.L., Palmiter, R.D., Hawrylycz, M.J., Jones, A.R., et al. (2010). A robust and high-throughput Cre reporting and characterization system for the whole mouse brain. Nat. Neurosci. *13*, 133–140.

Monnier, M., Fallert, M., and Battacharya, I.C. (1967). The waking action of histamine. Experientia *23*, 21–22.

Nagel, G., Brauner, M., Liewald, J.F., Adeishvili, N., Bamberg, E., and Gottschalk, A. (2005). Light activation of channelrhodopsin-2 in excitable cells of Caenorhabditis elegans triggers rapid behavioral responses. Curr. Biol. *15*, 2279–2284.

Nicholson, A.N., Pascoe, P.A., Turner, C., Ganellin, C.R., Greengrass, P.M., Casy, A.F., and Mercer, A.D. (1991). Sedation and histamine H1-receptor antagonism: studies in man with the enantiomers of chlorpheniramine and dimethindene. Br. J. Pharmacol. *104*, 270–276.

Panula, P., Yang, H.Y., and Costa, E. (1984). Histamine-containing neurons in the rat hypothalamus. Proc. Natl. Acad. Sci. USA *81*, 2572–2576.

Panula, P., Pirvola, U., Auvinen, S., and Airaksinen, M.S. (1989). Histamine-immunoreactive nerve fibers in the rat brain. Neuroscience *28*, 585–610.

Parmentier, R., Ohtsu, H., Djebbara-Hannas, Z., Valatx, J.L., Watanabe, T., and Lin, J.S. (2002). Anatomical, physiological, and pharmacological characteristics of histidine decarboxylase knock-out mice: evidence for the role of brain histamine in behavioral and sleep-wake control. J. Neurosci. *22*, 7695–7711.

Parmentier, R., Kolbaev, S., Klyuch, B.P., Vandael, D., Lin, J.S., Selbach, O., Haas, H.L., and Sergeeva, O.A. (2009). Excitation of histaminergic tuberomamillary neurons by thyrotropin-releasing hormone. J. Neurosci. *29*, 4471–4483.

Petreanu, L., Huber, D., Sobczyk, A., and Svoboda, K. (2007). Channelrhodopsin-2-assisted circuit mapping of long-range callosal projections. Nat. Neurosci. *10*, 663–668.

Pulver, S.R., Pashkovski, S.L., Hornstein, N.J., Garrity, P.A., and Griffith, L.C. (2009). Temporal dynamics of neuronal activation by Channelrhodopsin-2 and TRPA1 determine behavioral output in Drosophila larvae. J. Neurophysiol. *101*, 3075–3088.

Roberts, E., and Sherman, M.A. (1993). GABA–the quintessential neurotransmitter: electroneutrality, fidelity, specificity, and a model for the ligand binding site of GABAA receptors. Neurochem. Res. *18*, 365–376.

Root, D.H., Mejias-Aponte, C.A., Zhang, S., Wang, H.L., Hoffman, A.F., Lupica, C.R., and Morales, M. (2014). Single rodent mesohabenular axons release glutamate and GABA. Nat. Neurosci. *17*, 1543–1551.

Sakai, K., Takahashi, K., Anaclet, C., and Lin, J.S. (2010). Sleep-waking discharge of ventral tuberomammillary neurons in wild-type and histidine decarboxylase knock-out mice. Front. Behav. Neurosci. *4*, 53.

Saper, C.B., Fuller, P.M., Pedersen, N.P., Lu, J., and Scammell, T.E. (2010). Sleep state switching. Neuron *68*, 1023–1042.

Schöne, C., and Burdakov, D. (2012). Glutamate and GABA as rapid effectors of hypothalamic ‘‘peptidergic’’ neurons. Front. Behav. Neurosci. *6*, 81.

Schöne, C., Apergis-Schoute, J., Sakurai, T., Adamantidis, A., and Burdakov, D. (2014). Coreleased orexin and glutamate evoke nonredundant spike outputs and computations in histamine neurons. Cell Rep. *7*, 697–704.

Senba, E., Daddona, P.E., Watanabe, T., Wu, J.Y., and Nagy, J.I. (1985). Coexistence of adenosine deaminase, histidine decarboxylase, and glutamate decarboxylase in hypothalamic neurons of the rat. J. Neurosci. *5*, 3393–3402.

Shabel, S.J., Proulx, C.D., Piriz, J., and Malinow, R. (2014). Mood regulation. GABA/glutamate co-release controls habenula output and is modified by antidepressant treatment. Science *345*, 1494–1498.

Srinivas, S., Watanabe, T., Lin, C.S., William, C.M., Tanabe, Y., Jessell, T.M., and Costantini, F. (2001). Cre reporter strains produced by targeted insertion of EYFP and ECFP into the ROSA26 locus. BMC Dev. Biol. *1*, 4.

Stegmeier, F., Hu, G., Rickles, R.J., Hannon, G.J., and Elledge, S.J. (2005). A lentiviral microRNA-based system for single-copy polymerase II-regulated RNA interference in mammalian cells. Proc. Natl. Acad. Sci. USA *102*, 13212–13217.

Sundvik, M., and Panula, P. (2012). Organization of the histaminergic system in adult zebrafish (Danio rerio) brain: neuron number, location, and cotransmitters. J. Comp. Neurol. *520*, 3827–3845.

Takagi, H., Morishima, Y., Matsuyama, T., Hayashi, H., Watanabe, T., and Wada, H. (1986). Histaminergic axons in the neostriatum and cerebral cortex of the rat: a correlated light and electron microscopic immunocytochemical study using histidine decarboxylase as a marker. Brain Res. *364*, 114–123.

Takahashi, K., Lin, J.S., and Sakai, K. (2006). Neuronal activity of histaminergic tuberomammillary neurons during wake-sleep states in the mouse. J. Neurosci. *26*, 10292–10298.

Takeda, N., Inagaki, S., Shiosaka, S., Taguchi, Y., Oertel, W.H., Tohyama, M., Watanabe, T., and Wada, H. (1984). Immunohistochemical evidence for the coexistence of histidine decarboxylase-like and glutamate decarboxylase-like immunoreactivities in nerve cells of the magnocellular nucleus of the posterior hypothalamus of rats. Proc. Natl. Acad. Sci. USA *81*, 7647–7650.

Tong, Q., Ye, C.P., Jones, J.E., Elmquist, J.K., and Lowell, B.B. (2008). Synaptic release of GABA by AgRP neurons is required for normal regulation of energy balance. Nat. Neurosci. *11*, 998–1000.

Torrealba, F., Riveros, M.E., Contreras, M., and Valdes, J.L. (2012). Histamine and motivation. Front. Syst. Neurosci. *6*, 51.

Tritsch, N.X., Ding, J.B., and Sabatini, B.L. (2012). Dopaminergic neurons inhibit striatal output through non-canonical release of GABA. Nature *490*, 262–266.

Tritsch, N.X., Oh, W.J., Gu, C., and Sabatini, B.L. (2014). Midbrain dopamine neurons sustain inhibitory transmission using plasma membrane uptake of GABA, not synthesis. eLife *3*, e01936.

Trottier, S., Chotard, C., Traiffort, E., Unmehopa, U., Fisser, B., Swaab, D.F., and Schwartz, J.C. (2002). Co-localization of histamine with GABA but not with galanin in the human tuberomamillary nucleus. Brain Res. *939*, 52–64.

Tye, K.M., and Deisseroth, K. (2012). Optogenetic investigation of neural circuits underlying brain disease in animal models. Nat. Rev. Neurosci. *13*, 251–266.

Vaaga, C.E., Borisovska, M., and Westbrook, G.L. (2014). Dual-transmitter neurons: functional implications of co-release and co-transmission. Curr. Opin. Neurobiol. *29*, 25–32.

van den Pol, A.N. (2012). Neuropeptide transmission in brain circuits. Neuron *76*, 98–115.

Vincent, S.R., Hökfelt, T., Skirboll, L.R., and Wu, J.Y. (1983). Hypothalamic gamma-aminobutyric acid neurons project to the neocortex. Science *220*, 1309–1311.

Vong, L., Ye, C., Yang, Z., Choi, B., Chua, S., Jr., and Lowell, B.B. (2011). Leptin action on GABAergic neurons prevents obesity and reduces inhibitory tone to POMC neurons. Neuron *71*, 142–154.

Wada, H., Inagaki, N., Yamatodani, A., and Watanabe, T. (1991). Is the histaminergic neuron system a regulatory center for whole-brain activity? Trends Neurosci. *14*, 415–418.

Watanabe, T., Taguchi, Y., Hayashi, H., Tanaka, J., Shiosaka, S., Tohyama, M., Kubota, H., Terano, Y., and Wada, H. (1983). Evidence for the presence of a histaminergic neuron system in the rat brain: an immunohistochemical analysis. Neurosci. Lett. *39*, 249–254.

Williams, R.H., Chee, M.J., Kroeger, D., Ferrari, L.L., Maratos-Flier, E., Scammell, T.E., and Arrigoni, E. (2014). Optogenetic-mediated release of histamine reveals distal and autoregulatory mechanisms for controlling arousal. J. Neurosci. *34*, 6023–6029.

Wisden, W., Cope, D., Klausberger, T., Hauer, B., Sinkkonen, S.T., Tretter, V., Lujan, R., Jones, A., Korpi, E.R., Mody, I., et al. (2002). Ectopic expression of the GABA(A) receptor alpha6 subunit in hippocampal pyramidal neurons produces extrasynaptic receptors and an increased tonic inhibition. Neuropharmacology *43*, 530–549.

Wlodarczyk, A.I., Xu, C., Song, I., Doronin, M., Wu, Y.W., Walker, M.C., and Semyanov, A. (2013). Tonic GABAA conductance decreases membrane time constant and increases EPSP-spike precision in hippocampal pyramidal neurons. Front. Neural Circuits *7*, 205.

Wojcik, S.M., Katsurabayashi, S., Guillemin, I., Friauf, E., Rosenmund, C., Brose, N., and Rhee, J.S. (2006). A shared vesicular carrier allows synaptic corelease of GABA and glycine. Neuron *50*, 575–587.

Yu, X., Zecharia, A., Zhang, Z., Yang, Q., Yustos, R., Jager, P., Vyssotski, A.L., Maywood, E.S., Chesham, J.E., Ma, Y., et al. (2014). Circadian factor BMAL1 in histaminergic neurons regulates sleep architecture. Curr. Biol. *24*, 2838–2844.

Zecharia, A.Y., Yu, X., Götz, T., Ye, Z., Carr, D.R., Wulff, P., Bettler, B., Vyssotski, A.L., Brickley, S.G., Franks, N.P., and Wisden, W. (2012). GABAergic inhibition of histaminergic neurons regulates active waking but not the sleep-wake switch or propofol-induced loss of consciousness. J. Neurosci. *32*, 13062–13075.

Neuron, Volume *87*

Supplemental Information

Wakefulness Is Governed by GABA and Histamine Cotransmission

Xiao Yu, Zhiwen Ye, Catriona M. Houston, Anna Y. Zecharia, Ying Ma, Zhe Zhang, David S. Uygun, Susan Parker, Alexei L. Vyssotski, Raquel Yustos, Nicholas P. Franks, Stephen G. Brickley, and William Wisden

Supplementary Items

Figure S1 (related to Figure 3) shows the striatum-based turning assay used to test the efficacy of AAV constructs that knockdown or delete vGAT in striatum.

Figure S2 (related to Figures 3 and 4) shows the extent of *AAV-Cre-2A-Venus* expression in the TMN area resulting from bilateral injections.

Figure S3 (related to Figure 3F) shows qPCR on mRNA isolated from TMN tissue punches or single cells of *HDC-vgat KD* and *TMN-Δvgat* mice to assay the knockdown or deletion efficiency of *vgat* expression.

Figure S4 (related to Figures 3 and Figure 4) shows histamine levels in the neocortex and striatum of *HDC-vgat KD* and *TMN-Δvgat* mice.

Figure S5 (related to Figure 4) shows examples of EEG and EMG data used for scoring vigilance states and the time mice spent in each state.

Figure S6 (related to Figure 4) shows how the *HDC-vgat KD* and *TMN-Δvgat* mice responded to sleep deprivation.

Experimental Procedures

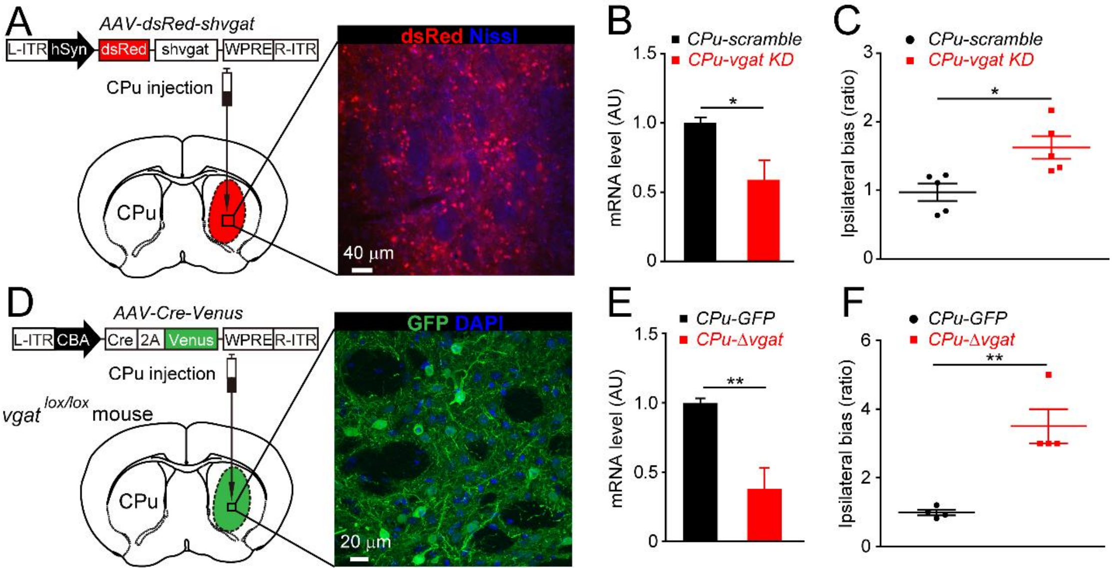

Figure S1 Striatal-based motor turning to check efficacy of the AAV constructs that knockdown or delete *vgat* expression in striatum. *Vgat* shRNA knockdown in the CPu was as effective as unilateral ablation of the *vgat* gene. Both the *CPu-vgat KD* and *CPu-Δvgat* mice had increased ipsilateral turning compared with controls.

(A) *AAV-dsRed-shvgat* and *AAV-dsRed-scramble* were unilaterally injected into the caudate putamen (CPu) of adult C57Bl6 mice to make *CPu-vgat KD* and *CPu-scramble* mice, respectively. Four weeks after injection, dsRed expression was visible in the CPu. Background: the CPu contains mainly GABAergic (medium spiny) projection neurons. When striatal function is unilaterally perturbed, animals tend to turn predominantly in one direction. (B) Q-PCR of *vgat* gene expression from CPu tissue punches shows *AAV-dsRed-shvgat* injection decreased *vgat* transcript levels compared with *AAV-dsRed-scramble*-injected control mice (*CPu-scramble*: 1 ± 0.04 *vs. CPu-vgat KD*: 0.58 ± 0.14, t-test, *p < 0.05). (C) Turning behavior experiments were performed with a separate cohort of injected mice. Knocking down *vgat* expression by injecting ipsilaterally *AAV-dsRed-shvgat* into the CPu caused

ipsilateral turning bias in those animals. Error bars represent sem. (*CPu-scramble*: 0.97 ± 0.12*, vs. CPu-vgat KD*: 1.62 ± 0.16*,* t-test, *p < 0.05). Method: a camera was attached above an open box, and mice were put into the box for 30 min. Data were collected by counting the video for the first 1 min or 5 min. (D) *AAV-Cre-2A-Venus* or *AAV-GFP* was injected into the CPu of *vgat*$^{lox/lox}$ mice to make *CPu-Δvgat* and *CPu-GFP* mice, respectively. Four weeks after injection, Venus was expressed in the CPu as seen by staining with GFP antisera. (E) qPCR of *vgat* gene expression from a virus injected CPu punch showed *AAV-Cre-Venus* decreased *vgat* gene expression compared with *AAV-GFP* injected control mice (*CPu-GFP*: 1 ± 0.03 *vs. CPu-Δvgat*: 0.37 ± 0.15, t-test, **p < 0.01, Error bars, sem). (F) Locally knocking out the *vgat* gene by injecting *AAV-Cre-2A-Venus* ipsilaterally into a portion of the CPu of *vgat*$^{lox/lox}$ mice caused an ipsilateral turning bias in those animals (*CPu-GFP*: 0.99 ± 0.08 *vs. CPu-Δvgat*: 3.5 ± 0.5, t-test, **p < 0.01).

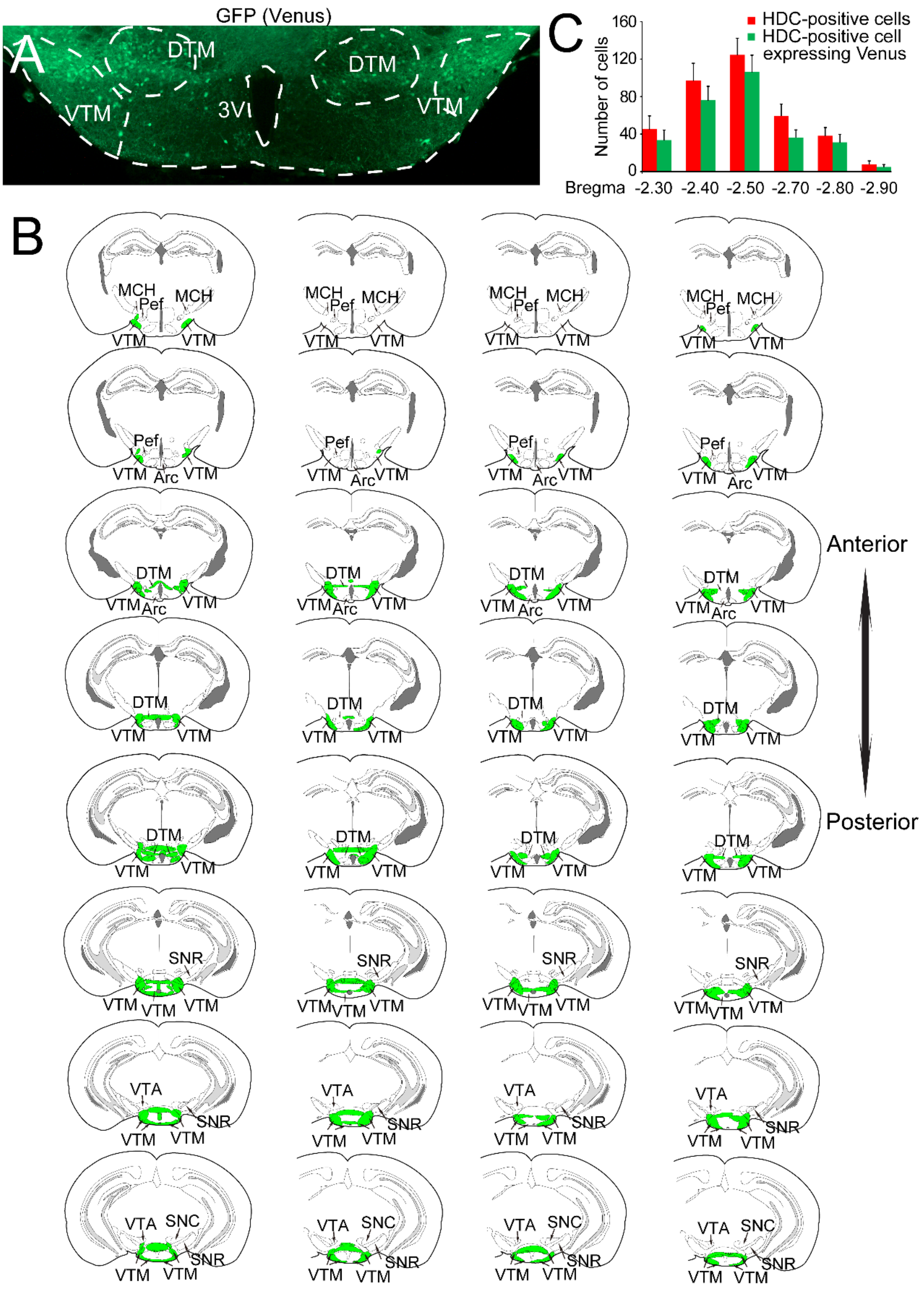

Figure S2 Mapping *AAV-Cre-2A-Venus* expression in the TMN area of *vgat*$^{lox/lox}$ mice (*TMN-Δvgat* mice). (A) Coronal section with bilateral *AAV-Cre-2A-Venus* injection shows Venus (GFP) expression in the VTM and DTM

area of the TMN. (B) Illustration of AAV transgene expression from 4 mice. Whole-brain sections were incubated with GFP antisera. GFP expression in soma was observed (as shown in A) and illustrated with green shading. Most of the GFP positive cells were restricted to the TMN area. A few GFP-cells were observed in the Pef area (n = 5 ± 2 cells; n = 4 mice) and in the substantia nigra reticulata (SNR) area (n = 4 ± 2 cells; n = 4 mice). Error bars, sem. (C) All brain sections were stained with HDC and GFP antisera and the HDC-positive and GFP-positive cells were counted. Most HDC-positive neurons were transduced by *AAV-Cre-2A-Venus* as determined by GFP expression (77 ± 2 %, n = 4 mice). Error bars, sem.

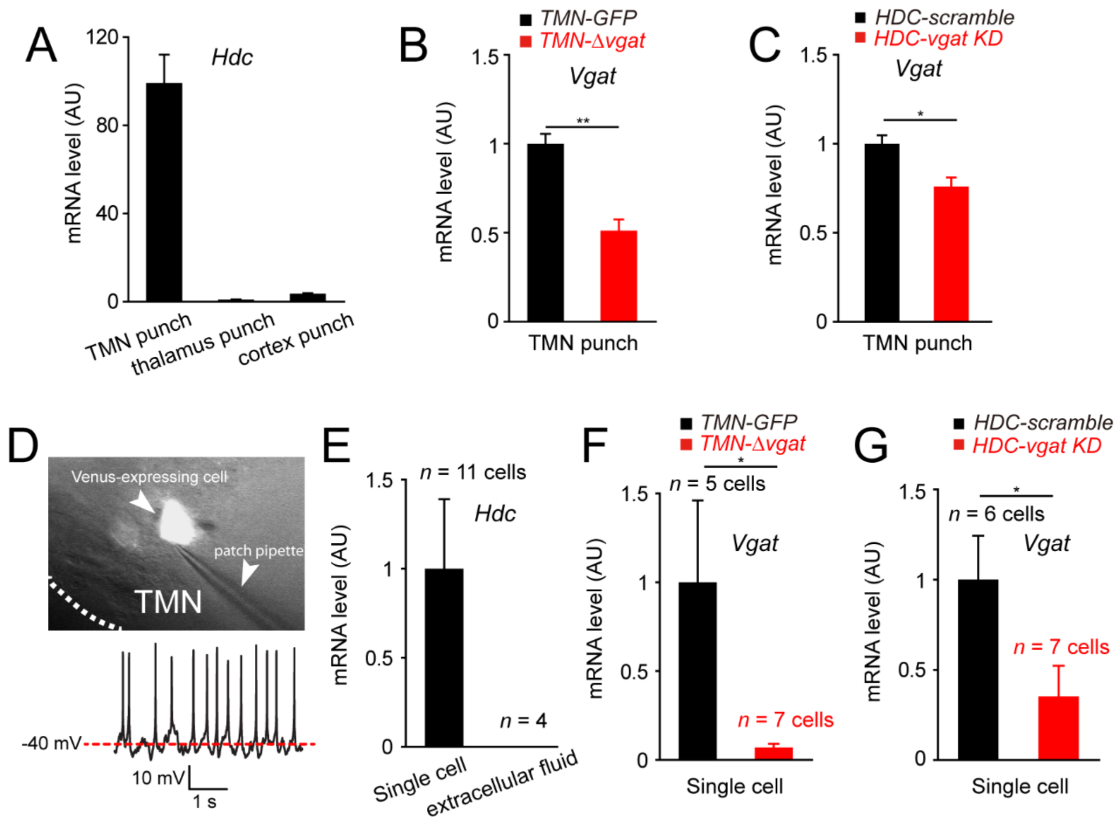

Figure S3 Testing efficiencies of deleting or knocking-down *vgat* expression in the TMN area of *TMN-Δvgat* and *HDC-vgat KD* mice. (A) positive control: qPCR on mRNA isolated from tissue punches from the TMN area showed that *hdc* was highly expressed in TMN punches, but not in thalamus and neocortex punches (n = 4 mice). (B, C) *vgat* mRNA levels were significantly decreased in the TMN punches of *TMN-Δvgat* (n = 4) and *HDC-vgat KD* (n = 4) mice compared with those from *TMN-GFP* (n = 4) and *HDC-scramble* (n = 4) mice (t-test, *p < 0.05, **p < 0.01). (D) An example of a Venus-expressing TMN cell from an acute brain slice of TMN from a *HDC-Δvgat* mouse. The neuron was patched and its firing (4 Hz) recorded. The patch pipette is visible. (E) Single-cell qPCR of *hdc* mRNA in some selected patched cells. *Hdc* mRNA was detected in patched TMN cells but not in extracellular fluid. In *HDC-scramble* and *HDC-vgat KD* mice, all the patched

cells were *hdc*-positive (13 out of 13 cells), because the fluorescence from AAV transduction is *HDC-Cre* dependent; in *TMN-Δvgat* and *TMN-GFP* mice, only 12 out of 16 cells patched were *hdc*-positive due to non-cell-type selective expression of the fluorescent AAV transduction (Cre-2A-Venus). (F, G) *vgat* mRNA levels were significantly decreased in the single patched cells from the TMN area of *TMN-Δvgat* (n = 7 cells) and *HDC-vgat KD* (n = 7 cells) mice compared with cells from *TMN-GFP* (n = 5 cells) and *HDC-scramble* (n = 6 cells) mice (t-test, *$p < 0.05$). Error bars, sem.

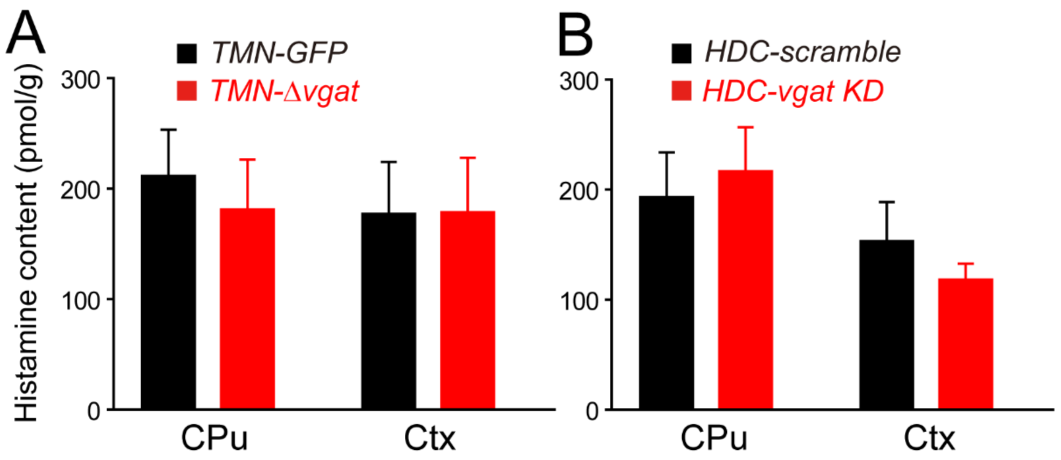

Figure S4 Histamine level in the neocortex or striatum of *HDC-vgat KD* and *TMN-Δvgat* mice. (A) Histamine levels in the striatum or neocortex of *TMN-GFP* (*n* = 6) and *TMN Δvgat* (*n* = 5) mice (t-test, $p > 0.05$, bars, sem). (B) Histamine levels in the striatum or neocortex of *HDC-scramble* (*n* = 5) and *HDC-vgat KD* (*n* = 4) mice (t-test, $p > 0.05$, bars, sem).

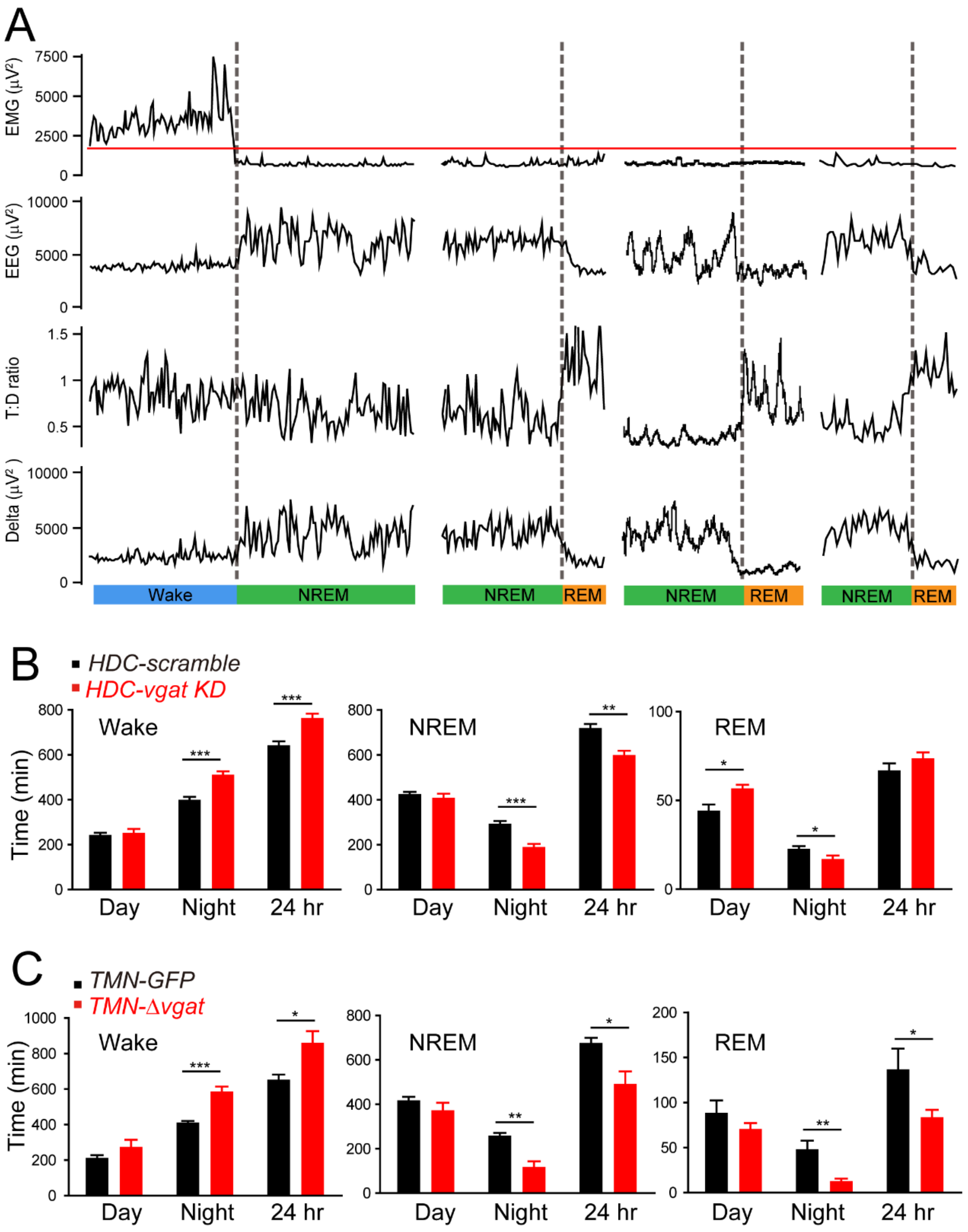

Figure S5 EEG scoring and additional sleep-wake parameters from *HDC-vgat KD* and *TMN-Δvgat* mice. (A) Example traces of EMG and EEG power during wake, NREM, and REM from the mice. T:D ratio, theta/delta ratio. (B) Time of wake, NREM and REM sleep during the day (0-12 h), night (12-24 h) and entire 24 hr of *HDC-scramble* (*n* = 6) and *HDC-vgat KD* (*n* = 6) mice (t-test, *p < 0.05, **p < 0.01, ***p < 0.001, bars, sem). (C) Time of wake, NREM

and REM sleep during the day (0-12 h), night (12-24 h) and entire 24 hr of *TMN-GFP* (n = 5) and *TMN-Δvgat* (n = 7) mice (t-test, *$p < 0.05$, **$p < 0.01$, ***$p < 0.001$, bars, sem).

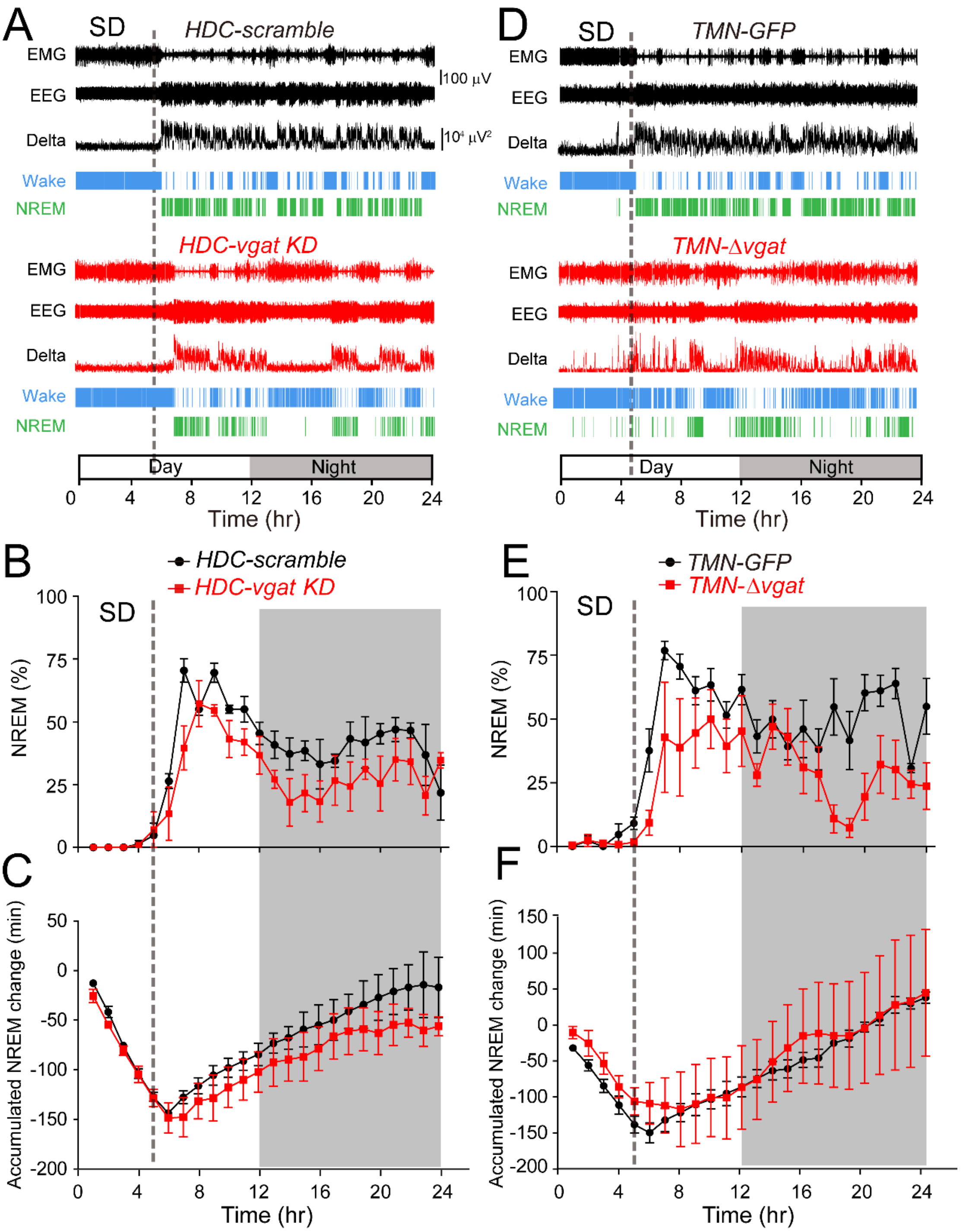

Figure S6. Responses to sleep deprivation of *HDC-vgat KD* and *TMN-Δvgat* mice. (A) Continuous EMG, EEG, delta power, wake, NREM sleep and REM sleep scoring data recorded for an *HDC-scramble* and an *HDC-vgat KD* mouse during and after sleep deprivation (SD). The period where SD ends is marked by the perpendicular dotted gray line. (B) Percentage of NREM sleep

during 5-hr sleep deprivation and following 19-hr recovery sleep of *HDC-scramble* (*n* = 4) and *HDC-vgat KD* (*n* = 4) mice. Bars, sem. (C) The accumulated NREM change compared with the normal 24-hr sleep-wake cycle. The proportional loss and recovery of NREM sleep in minutes during and after sleep deprivation of *HDC-scramble* (*n* = 4) and *HDC-vgat KD* (*n* = 4) mice. Bars, sem. (D) Continuous EMG, EEG, delta power, wake, NREM sleep and REM sleep scoring data recorded for a *TMN-GFP* and *TMN-Δvgat* mouse during and after sleep deprivation (SD). The period where SD ends is marked by the perpendicular dotted gray line. (E) Percentage of NREM sleep during 5-hr sleep deprivation and following 19-hr recovery sleep of *TMN-GFP* (*n* = 5) and *TMN-Δvgat* (*n* = 4) mice. Bars, sem. (F) The accumulated NREM change compared with the normal 24-hr sleep-wake cycle. The proportional loss and recovery of NREM sleep in minutes during and after sleep deprivation of *TMN-GFP* (*n* = 5) and *TMN-Δvgat* (*n* = 4) mice. Bars, sem.

Experimental Procedures

Mouse genetics

To generate the *HDC-YFP* mice, *HDC-Cre* mice were crossed with *Rosa26-loxP-Stop-loxP-YFP* mice. To generate the *vGAT-tdTomato* mice, *vGAT-Cre* mice were crossed with *Rosa26-loxP-Stop-loxP-tdTomato* mice. Genotyping primers were:

***iCre* (*HDC-Cre*)**:
forward: 5'GTGTGGCTGCCCCTTCTGCC3',
reverse: 5'AGCCTCACCATGGCCCCAGT3' (250 bp product);

***vgat*$^{lox/lox}$**:
common: 5'TCCTTTGTGGCTTCCTTCCG3',
WT reverse: 5'GGATAGAAGAAGTGTGGACC3',
Mut reverse: 5'GCAGTGGACCTTGGATGTCTATC3' (WT: 185 bp, Mut: 250 bp);

Rosa-YFP:
forward:5'GGCGCTACCGGTCGCCACCATGGTGAGCAAGGGCGAGGAGC 3',
reverse: 5'GCGCGCGTTAACTTACTTGTACAGCTCGTCCATGCC3' (980 bp);

vGAT-Cre:
forward: 5'AGCGATGGATTTCCGTCTCT3',
reverse: 5'CACCAGCTTGCATGATCTCC3' (200 bp)

Rosa-tdTomato:
WT forward: 5'AGGGGAGCTGCAGTGGAGTA3',
WT reverse: 5'CCGAAAATCTGTGGGAAGTC3';
Mut forward: 5'GGCATTAAAGCAGCGTATCC3',
Mut reverse: 5'CTGTTCCTGTACGGCATGG3' (WT: 297 bp, Mut: 196 bp).

Design and testing of *shvgat* expression vectors

Hairpin (*sh*) oligonucleotides were designed online (http://katahdin.mssm.edu/siRNA/RNAi.cgi?type=shRNA). The *sh* oligonucleotide sequences tested were:

scramble: 5'-TGCTGTTGACAGTGAGCGGCCGCGATTAGGCTGTTATAATAGTGAAGCCACAGATGTATTATAACAGCCTAATCGCGGCTGCCTACTGCCTCGGA-3'

shvgat1: 5'-TGCTGTTGACAGTGAGCGCGGTGTGCTCGTGGTGAATAAGTAGTGAAGCCACAGATGTACTTATTCACCACGAGCACACCATGCCTACTGCCTCGGA-3'

shvgat2: 5'-TGCTGTTGACAGTGAGCGAAGCCATTCAGTGCTTGGAATCTAGTGACCACAGATGTAGATTCCAAGCACTGAATGGCTGTGCCTACTGCCTCGGA-3'

shvagt3: 5'-TGCTGTTGACAGTGAGCGCACTCATCTTGTGCAATGTATCTAGTGAAGCCACAGATGTAGATACATTGCACAAGATGAGTTTGCCTACTGCCTCGGA-3'

The *sh*-oligonucleotides were amplified by VENT polymerase (NEB, UK) using primers with added XhoI and EcoRI sites underlined (forward: 5'-GATGGCTG-CTCGAG-AAGGTATAT-TGCTGTTGACAGTGAGCG-3'; reverse: 5'-GTCTAGAG-GAATTC-CGAGGCAGTAGGCA-3'). Products were cloned into *the EcoRI/XhoI* site of the pPRIME-CMV-dsRed-FF3 vector (Stegmeier et al., 2005) to generate *pPRIME-dsRed-scramble*, *pPRIME-dsRed-shvgat1* (oligo 1), *pPRIME-dsRed-shvgat2* (oligo 2) and *pPRIME-dsRed-shvgat3* (oligo 3) (Figure 3A). The *pPRIME-CMV-dsRed-FF3* vector was a gift from Stephen Elledge (Addgene plasmid 11664).

To construct the *vgat* expression vector, the mouse *vgat* coding sequence and 3' UTR was PCR amplified with primers (forward: 5'-ATATAT-GCTAGC-CCCAGACCCTTCTGTCCTTTTCTC-3'; reverse: 5'-ATATAT-AAGCTT-CGCCTTTGTTTCTTCTTTATTTGCGG-3') with *HindIII* and *NheI* sites from a mouse *vgat* cDNA clone (Clontech, CA). The digested PCR insert was purified and cloned into the *pcDNA3.1* expression vector. The *vgat* expression plasmid was co-transfected into HEK293 cells with either *pPRIME-dsRed-shvgat1, pPRIME-dsRed-shvgat2, pPRIME-dsRed-shvgat3 or pPRIME-dsRed-scramble*. 24-36 hours after transfection, cells were fixed with paraformaldehyde, and stained with vGAT antisera (see section "Immunocytochemistry" below); dsRed was visualized by primary fluorescence. Using this method, we established that *shvgat* oligo3 was

effective. The *shvgat* and *scramble* segments were then placed into AAV genomes (see next section).

To generate the *AAV-dsRed-scramble* and *AAV-dsRed-shvgat* transgenes, the *pPRIME-dsRed-scramble* and *pPRIME-dsRed-shvgat* vectors were PCR amplified using the primers (forward: 5'-AGTGAACCGTCGGCGCGCCTAGCGCTACC-3'; reverse: 5'-CGTGTTTAAACGCGCTAGCCTTCCAATTG-3') with an *AscI* site in the forward primer and a *NheI* site in the reverse primer. PCR products, and were digested with *AscI* and *NheI*, and cloned into an AAV vector with a human synapsin promoter. To construct *AAV-flex-dsRed-scramble* and *AAV-flex-dsRed-shvgat* plasmids, *pPRIME-dsRed-scramble* and *pPRIME-dsRed-shvgat* were PCR amplified using primers (forward: 5'-GAACCGTCAGATCCGCTAGCGCTACCG-3'; reverse: 5'-GAACGCGTCGGCGCGCCTTTAAACGCAT-3') with a *NheI* site in the forward primer and an *AscI* site in the reverse primer. PCR products and AAV vector were digested with *AscI* and *NheI*, and cloned into the AAV vector. The AAV transgenes were then packaged into capsids (see Experimental procedures in the main manuscript).

Histology

For immunocytochemistry, mice were transcardially perfused with 4% paraformaldehyde in phosphate buffered saline (PBS). Brains were removed and 35-µm-thick coronal sections were cut using a Leica VT1000S vibratome. Free-floating sections were washed in PBS three times for 5 min, permeabilized in PBS plus 0.4% Triton X-100 for 30 min, blocked by incubation in PBS plus 5% normal goat serum (NGS) (Vector), 0.2% Triton X-100 for 1 hour (all at room temperature) and subsequently incubated with primary antisera or antibody diluted in PBS plus 2% NGS for 24-48 hours at 4°C on a shaker. Incubated slices were washed three times in PBS for 10 min at room temperature, and then incubated for 2 hours at room temperature with a 1:1000 dilution of a secondary antibody (Molecular Probes) in PBS plus 1% NGS, and subsequently washed there times in PBS for 10 min at room temperature. For cell cultures, medium was removed and cells were washed

in PBS three times for 5 min, and then fixed in PFA for 10 min, after that, cells were permeabilized in PBS plus 0.4% Triton X-100 for 10 min; blocked by incubation in PBS plus 5% normal goat serum (NGS) (Vector), 0.2% Triton X-100 for 10 min (all at room temperature) and subsequently incubated with primary antisera or antibody diluted in PBS plus 2% NGS, shaken overnight at 4°C. Incubated cells were washed three times in PBS for 10 min at room temperature, and then incubated for 2 hours at room temperature with a 1:1000 dilution of a secondary antibody (Molecular Probes) in PBS plus 1% NGS, and subsequently washed there times in PBS for 10 min at room temperature. Primary antisera or antibody used were: rabbit polyclonal EGFP (Invitrogen Molecular Probes, Eugene, OR), 1:1000; guinea pig polyclonal HDC (American Research Products, Belmont, MA), 1:300; mouse monoclonal GAD67 (Millipore, UK), 1:1000; mouse monoclonal vGAT (Synaptic Systems), 1:1000. Secondary antibodies were Alexa Fluor 488 goat anti-rabbit IgG, 1:1000, Alexa Fluor 488 goat anti-mouse IgG, 1:1000; Alexa Fluor 488 goat anti-guinea pig IgG, 1:1000, Alexa Fluor 594 goat anti-rabbit IgG, 1:1000, Alexa Fluor 594 goat anti-mouse IgG, 1:1000; Alexa Fluor 594 goat anti-guinea pig IgG, 1:1000, (Invitrogen Molecular Probes); Slices were mounted on slides, embedded in Mowiol (DAPI or without DAPI), cover-slipped, and analyzed using a Zeiss LSM 510 confocal microscope (Facility for Imaging by Light Microscopy, Imperial College).

Imaging neurons after electrophysiology: Following electrophysiological recording (see section “Electrophysiology”), brain slices were fixed in 4% PFA, permeabilized with 0.2% Triton-X at room temperature for 1-2 hours, and non-specific binding blocked with 5% donkey serum in PBS. A 1:200 dilution of streptavidin-Alexa555 (Invitrogen Molecular Probes) was then conjugated to the biocytin and slices were washed in PBS and mounted in Vectashield® H-1000. Individual neurons were imaged using a Zeiss LSM 510 confocal microscope (Facility for Imaging by Light Microscopy, Imperial College).

EEG analysis and sleep-wake behavior

Surgery was carried out under halothane (1.5–2.5% in oxygen) anesthesia. Three gold-plated EEG electrodes (Decolletage AG) were inserted through

the skull onto the dura mater. For EMG recording, three lengths of Teflon-insulated stainless steel wire were inserted in the neck muscle. Animals were allowed at least 14 days to recover from surgery. Two days before the recording, mice were attached with mock Neurologgers, and then fitted with Neurolgger2A devices. Two EEG and two EMG channels for each mouse were recorded. Spike2 (version 7.10) was used to analysis the sleep (EEG/EMG) data. The sampling rate was set up to 200 Hz. EMG was filtered by band pass between 5 and 45 Hz. EEG frequency was filtered below 0.5 Hz. EEG power was analysed using FFT (Fast Fourier transform of the autocorrelation function) power spectra (FFT size: 1024 or 512).

Sleep deprivation

Mice were attached with mock Neurologgers two days before sleep deprivation. Before sleep deprivation, mice were attached with Neurologger 2A devices. Mice were sleep deprived for 5 hours after ‘‘lights off’’. In the first hour of the sleep deprivation (at the beginning of lights on), mice were put into novel cages with new objects. At each hour, objects were changed with new objects. During the last two hours, mice were gently handled for some seconds when they tended to sleep. Most of the time during the last two hours, mice were active and in a total of two hours, the mice only needed to be handled one or two times. After sleep deprivation, the mice were put back into their home cage to recover.

Electrophysiology

Adult (3-6 months postnatal) mice were handled to reduce their stress, and brain slices were then prepared following cervical dislocation (in accordance with UK Home Office guidelines). The brain was rapidly removed and immersed in ice cold slicing solution. The slicing solution contained (in mM): NaCl 85, KCl 2.5, $CaCl_2$ 0.5, MgCl 3.5, NaH_2PO4 1.25, $NaHCO_3$ 26, D-glucose 11, sucrose 75, 1 kynurenic acid) pH 7.4 when bubbled with 95%O2/5%CO2. Slices were cut using a vibratome tissue slicer (Campden instruments) at a thickness of 250 µm and immediately transferred to a holding chamber containing slicing ACSF continuously bubbled with

95%O2/5%CO2. Once slicing was complete the holding chamber was transferred to a 37°C heat block for 40 minutes, after which the slicing ACSF was gradually exchanged for recording ACSF (in mM: NaCl 125, KCl 2.5, CaCl2 2, MgCl 1, NaH_2PO_4 1.25, $NaHCO_3$ 26, glucose 11, pH 7.4 when bubbled with 95%O2/5%CO2) and allowed to reach room temperature while the solutions were exchanged prior to electrophysiological recording. Slices were visualized using a fixed-stage upright microscope (BX51W1, Olympus and Scientifica Slice scope) fitted with a high numerical aperture water-immersion objective and an infra-red sensitive digital camera. For the optogenetic experiments with *HDC-ChR and HDC-ChR/TMN-Δvgat* mice, we first sliced the TMN area. If there was visible primary fluorescence in this area (e.g. Fig. 6B), we also went on to slice the neocortex and caudate-putamen. Some TMN injections gave no visible primary expression in the TMN and slices from these brains were used as negative controls. A blue (470 nm) collimated LED (M470L3-C1, Thorlabs) was used to illuminate the slice through the objective lens. The LED was driven by an LED-driver (LEDD1B, Thorlabs) which was controlled by a National Instruments digitization board (NI-DAQmx, PCI-6052E; National Instruments, Austin, Texas). The output of the LED was measured with a power meter (PM100D, Thorlabs) positioned below the objective lens and the power output and membrane conductance or voltage changes were aligned for analysis.

Patch pipettes were fabricated from thick-walled borosilicate glass capillaries (1.5 mm outer diameter., 0.86 mm internal diameter, Harvard Apparatus) using a two-step vertical puller (Narishige, PC-10). Pipette resistances were typically 3-4 MΩ when back filled with internal solution. For voltage-clamp experiments, the internal solution contained (in mM): CsCl 140, NaCl 4, $CaCl_2$ 0.5, HEPES 10, EGTA 5, Mg-ATP 2; the pH was adjusted to 7.3 with CsOH. Biocytin (1.5 mg/ml) was included in the pipette solution so that neuronal cell-type could be confirmed. For current-clamp experiments the internal solution contained (in mM): 145 K-gluconate; 4 NaCl; 0.5 $CaCl_2$; 10 HEPES; 5 EGTA; 4 Mg-ATP; 0.3 Na-GTP (adjusted to pH 7.3 with KOH). The amplifier head

stage was connected to an Axopatch 700B amplifier (Molecular Devices; Foster City, CA).

Electrophysiology data analysis

The amplifier current output was filtered at 10 kHz (–3 dB, 8-pole low-pass Bessel) and digitized at 20 kHz using a National Instruments digitization board (NI-DAQmx, PCI-6052E; National Instruments, Austin, Texas). Data acquisition was performed using WINWCP (Version 4.1.2) and WINEDR (Version 3.0.9) kindly provided by John Dempster (University of Strathclyde, UK). The initial rising phase of the spontaneous postsynaptic conductance change (sPSC) was detected on the first derivative of the current record obtained at a command potential of -70 mV. The instantaneous sPSC frequency was calculated from the inter sPSC interval and the running average was calculated every 5 seconds. Cross correlation analysis was undertaken on the timing of PSCs using a 100 ms sliding window before and after each LED pulse. The change in holding current was plotted from the average of each one second current epoch. Any change in the tonic $GABA_A$ receptor meditated conductance (G_{tonic}) was calculated from changes in holding current averaged over at least one minute.

Negative controls for light stimulation: some AAV injections into the TMN area of *HDC-Cre* mice missed the target, and so there was no ChR-YFP expression detected by primary fluorescence when we sliced the brains. Slices from these mice provided negative controls for light stimulation in the neocortex and caudate-putamen: the steady-state holding current recorded from layer IV cortical neurons with no ChR expressed in the TMN was -320 ± 140 pA before and -300 ± 130 pA after 5Hz light stimulation, demonstrating no significant change (reduction of -0.24 ± 0.13 nS; n = 7) in G_{tonic} (paired t-test, p = 0.1). For caudate-putamen slices with no *HDC-ChR-YFP* expression, on average there was also a 0.3 ± 0.1 nS (n = 4) decrease in G_{tonic} during the 5 Hz light stimulation.
